# Developing quality indicators for cross-sectoral psycho-oncology in Germany: combining the RAND/UCLA appropriateness method with a Delphi technique

**DOI:** 10.1186/s12913-023-09604-3

**Published:** 2023-06-08

**Authors:** Lisa Derendorf, Stephanie Stock, Dusan Simic, Clarissa Lemmen

**Affiliations:** Institute for Health Economics and Clinical Epidemiology (IGKE), University of Cologne, Faculty of Medicine and University Hospital Cologne, Gleueler Str. 176-178, 50935 Cologne, Germany

**Keywords:** Quality measurement, Quality indicator, Quality improvement, Quality improvement methodologies, RAND/UCLA Appropriateness Method, Delphi Technique, Psycho-oncology, Cancer, Complex intervention, Integrated care model

## Abstract

**Background:**

Internationally, the need for appropriately structured, high-quality care in psycho-oncology is more and more recognized and quality-oriented care is to be established. Quality indicators are becoming increasingly important for a systematic development and improvement of the quality of care. The aim of this study was to develop a set of quality indicators for a new form of care, a cross-sectoral psycho-oncological care program in the German health care system.

**Methods:**

The widely established RAND/UCLA Appropriateness Method was combined with a modified Delphi technique. A systematic literature review was conducted to identify existing indicators. All identified indicators were evaluated and rated in a two-round Delphi process. Expert panels embedded in the Delphi process assessed the indicators in terms of relevance, data availability and feasibility. An indicator was accepted by consensus if at least 75% of the ratings corresponded to category 4 or 5 on a five-point Likert scale.

**Results:**

Of the 88 potential indicators derived from a systematic literature review and other sources, 29 were deemed relevant in the first Delphi round. After the first expert panel, 28 of the dissented indicators were re-rated and added. Of these 57 indicators, 45 were found to be feasible in terms of data availability by the second round of expert panel. In total, 22 indicators were transferred into a quality report, implemented and tested within the care networks for participatory quality improvement. In the second Delphi round, the embedded indicators were tested for their practicability. The final set includes 16 indicators that were operationalized in care practice and rated by the expert panel as relevant, comprehensible, and suitable for care practice.

**Conclusion:**

The developed set of quality indicators has proven in practical testing to be a valid quality assurance tool for internal and external quality management. The study findings could contribute to traceable high quality in cross-sectoral psycho-oncology by providing a valid and comprehensive set of quality indicators.

**Trial registration:**

“Entwicklung eines Qualitätsmanagementsystems in der integrierten, sektorenübergreifenden Psychoonkologie—AP “Qualitätsmanagement und Versorgungsmanagement” zur Studie "integrierte, sektorenübergreifende Psychoonkologie (isPO)" a sub-project of the “integrierte, sektorenübergreifende Psychoonkologie (isPO)”, was registered in the German Clinical Trials Register (DRKS) (DRKS-ID: DRKS00021515) on 3rd September 2020. The main project was registered on 30th October 2018 (DRKS-ID: DRKS00015326).

**Supplementary Information:**

The online version contains supplementary material available at 10.1186/s12913-023-09604-3.

## Background

The incidence of cancer is increasing significantly worldwide [1, 2], with nearly 500,000 new cases diagnosed in Germany each year [3]. Cancer patients are affected by emotional distress and often by psychological disorders [4–9]. In Germany, the implementation of cross-sectoral psycho-oncological support is considered an important strategy to improve quality of cancer care for patients of all cancer entities. The German National Cancer Plan (NCP) strongly recommends to further develop "oncological care structures and quality assurance” [10, 11]. The implementation of psycho-oncological care structures along with quality management are not only complex and considered an integral part of oncology care [12, 13], but must also meet the demands for needs-based and accessible care, while being subjected to legally binding quality assurance terms [14].

Monitoring and improving quality in health care is of crucial importance, even if quality itself is not directly observable and measurable. Therefore, quality-related indicators are employed to make health care measurable [13, 14]. A quality indicator is a quantitative measure that can be used to monitor and assess the quality of governance and management, as well as clinical and support functions that impact patient outcomes in a process of care. They do not measure quality directly, but are rather a performance assessment tool that can draw attention to potential performance issues that may require more intense review within an organization [15]. Quality indicators have notably gained momentum because they systematically point out potential for improvement in a functioning quality management system [16, 17]. Indicators in health care are often applied for quality measurement and improvement (e.g., plan-do-check-act cycles (PDCA)), but also, for example, for comparison with other service providers (e.g., benchmarking), public disclosure (e.g., quality reports), or quality-based remuneration of services (pay for performance) as well as for research purposes [18–21].

To improve routine care of cancer patients in cancer centres in Germany, an intervention called “*new form of care integrated, cross-sectoral psycho-oncology”* (nFC-isPO) has been developed and piloted. In Germany, the health care system is divided into an inpatient and an outpatient sector. Treatment and diagnostics conducted during a hospital stay belong to the inpatient sector, whereas all treatment and rehabilitation activities outside of the hospital belong to the outpatient sector. “New forms of care” (nFC) are care models that improve cross-sectoral care, optimise intersectoral interfaces, or overcome the separation of sectors [22–24]. In the inpatient sector, psycho-oncological care is often provided in acute hospitals and oncological rehabilitation facilities. Although cancer counselling centres are well established in the outpatient sector, they will not be able to meet the demand in the medium term due to the demographic trends and the associated increase in the number of new cases [3, 25, 26]. A detailed examination of the psycho-oncological care structures in Germany by the Federal Ministry of Health (2018) showed that the degree of coverage of inpatient psycho-oncological care by psycho-oncological services in Germany can vary considerably depending on the sector and region. For example, more than half of the regions in the outpatient sector and about 40% in the inpatient sector have a coverage of less than 50% [27].

The nFC-isPO has bridged the gap from bench to bedside by providing a high quality, translational psycho-oncological care program for cancer patients [22, 28–31]. To ensure that care is delivered as stipulated, an appropriate and reliable set of quality indicators was needed for comprehensive quality management [22, 32]. The aim of this study was to develop, implement and evaluate a set of suitable indicators to systematically measure, manage, and improve the quality of care for a cross-sectoral psycho-oncological care program for cancer patients in routine care in several cancer centres in Germany.

## Methods

### Design

**Fig. 1:**
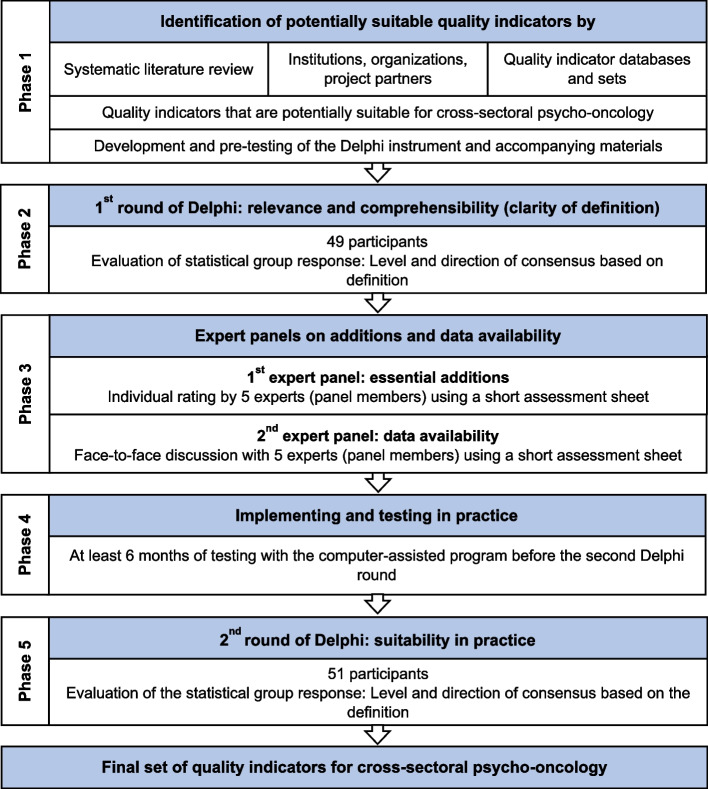
Modified process of developing quality indicators for cross-sectoral psycho-oncology

A procedure of linking the RAND/UCLA Appropriateness Method (RAM) with elements of the Delphi technique was used to develop a set of quality indicators to measure the quality of care regarding structures, processes, and outcomes of a cross-sectoral psycho-oncology care program [33, 34]. This methodology was useful to combine scientific evidence and expert opinion obtained through consensus technique. The iterative approach included a systematic literature review, a two-stage anonymous survey (Delphi rounds), a questionnaire-based reassessment of indicators and a face-to-face expert panel discussion (see Fig. 1) [17, 34, 35]. This project was registered in the German Clinical Trials Register (DRKS) (DRKS-ID: DRKS00021515) on 03/09/2020.

### Systematic literature search and selection of potential quality indicators

In June 2018, a systematic literature search was conducted to identify an initial set of quality indicators and domains of quality of care for cancer patients with emotional distress or mental disorder. Initially, six databases (PubMed, PsychINFO, Livivo, PSYNDEX, SpringerLink, Cochrane Library) were systematically searched for scientific articles. A predefined search strategy was used (see Additional file 1). In addition, bibliographies of relevant secondary publications and grey literature (e.g., reports on quality assurance projects), websites of relevant organizations that have developed or were using quality indicators (e.g., medical societies), and evidence-based guidelines recommending quality indicators were reviewed by hand search. The authors also identified indicators from the four care networks cooperating in the project. Study selection and screening were performed independently by two researchers (LD and CL). Duplicate indicators were removed (see Additional file 2). The identification of potential indicators was done by consensus between the two authors (LD and CL). Subsequently, the results were categorised based on Donabedian’s quality dimension (structure-, process- or outcome quality) [33, 36], and the recommended quality criteria of Joint Commission on Accreditation of Healthcare Organizations (JCAHO) (accessibility, appropriateness, continuity, efficiency, efficacy, patient perspective, safety, timeliness) [37, 38]. A preliminary set of indicators was selected to start the expert consensus and rating process (phase 1).

### Selection of survey participants and panel members

In a Delphi, the selection of the panel is based on the members' knowledge of the particular topic. Therefore, a purposive sampling strategy was was used to select the experts [39, 40]. The inclusion criterion was that the panel members had to be involved in the nFC-isPO team, as they had the background information on the development, implementation and testing of the nFC-isPO. The participants needed to be able to assess the project-specific requirements of the nFC-isPO for the development of indicators and for the availability of data. The Delphi rounds involved participants from different fields of health services research and psycho-oncological care who all operated in the care program (e.g., health care professionals, health insurance companies, patient representatives etc.). Although the first and second round of Delphi (phase 2 and 5) had a closed group of participants, participants who did not participate in the first round were allowed to take part in the second Delphi round (e.g., due to staff turnover) [41]. The multidisciplinary expert panel consisted of five nFC-isPO representatives from the fields of psycho-oncology, quality management, health services research and medical statistics.

### Rating the indicators

Based on the results of the literature search (phase 1), the survey items for the first Delphi round (phase 2) were developed and set up in the online survey tool “Limesurvey”, before being tested for functionality and comprehensibility. In order to assess the relevance and comprehensibility of the indicators, two assessment questions were developed for each indicator instead of a single global rating. At first, participants were asked to rate the relevance on a verbally named five-point Likert Scale (5 = relevant, 4 = rather relevant, 3 = partly relevant, 2 = rather not relevant, 1 = not relevant). Relevance was defined as the extent to which the characteristics of the indicator are appropriate for the concept being assessed [34]. Secondly, the authors asked for comprehensibility of the indicators, i.e. clarity of definition, by using a binary decision question (yes/no). Additional free-text options enabled the participants to comment on the need for change in definition or to suggest missing indicators based on their professional judgement. This structure allowed to consider specific adjustments when revising and optimizing the indicators in the following process. Phase 2 resulted in an overview of consented and dissented indicators. The results of the Delphi rounds were made available to the participants in the quality circles and the quality workshops.Based on the results of the first Delphi round, the expert panel evaluated the dissented indicators again individually with regard to relevant additions, taking special account of the free-text comments using an assessment sheet. The panel members then individually discussed and rated the operability and feasibility (i.e., data availability) of the preliminary indicator set using a short assessment sheet [42]. The face-to-face discussion took place at the “Centrum für Integrierte Onkologie (CIO)” at University Hospital of Cologne (phase 3). In phase 4, the indicators assessed as feasible were operationalised and systematically implemented into practice. The testing took place over a period of at least six months in four different health care networks.

The implemented and tested indicators were re-rated in the second Delphi round (phase 5) with regard to their practical suitability for assessing and managing the quality of care in the care program. In addition to the rating on the 5-point Likert scale, the participants had the opportunity to leave comments in a free-text box.

### Definition of consensus and statistical analysis

The consensus rule for assessing agreement and disagreement of the indicators in the Delphi process was established a priori. For descriptive statistical analysis, the authors used a proportion within a limited range [43]. The determined threshold of consensus is at 75% agreement, summed for categories 4 and 5 (agreement) or 1 and 2 (disagreement) [44].

- An indicator was considered to have a "moderate consensus" rating if the percentage of ratings of "relevant (5)" or "rather relevant (4)" ( +) reaches at least 75% consensus out of all valid responses.
- An indicator was considered to have a "strong consensus" rating if the percentage of ratings of "relevant (5)" or "rather relevant (4)" (+ +) reaches at least 90% consensus out of all valid responses.
- Evaluated as "moderate rejection" (-) if the proportion of evaluations with "not relevant (1)" and "rather not relevant (2)" reaches at least 75% consensus out of all valid responses.
- Evaluated as "strong rejection" (–) if the proportion of evaluations with "not relevant (1)" and "rather not relevant (2)" reaches at least 90% consensus out of all valid responses.
- All other indicators that had no unanimous group response (neither agreed nor disagreed), were considered dissent.

## Results

**Fig. 2:**
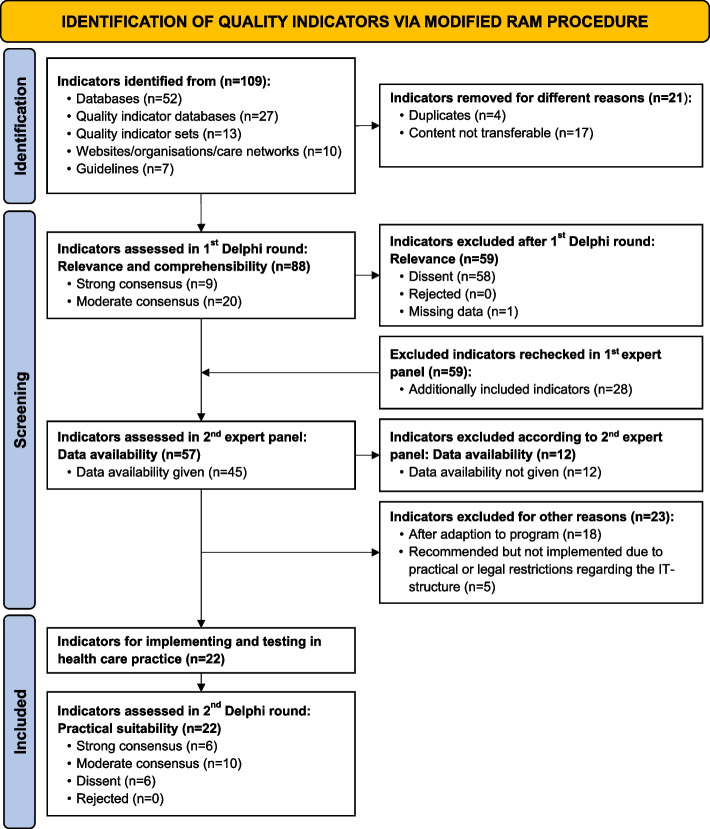
Results of the modified RAM procedure

An overview of the identified and evaluated indicators can be seen in Fig. 2.

### Participation in the study and characteristics of participants

For the first round of the Delphi, 49 participants were invited. Of the 49 participants in the first round (100% response rate), 27 properly completed the questionnaire (55% completion rate). 21 (41.2%) of the participants of the first round also participated in the second round. Here, especially the care network teams were asked to share the survey in-house with the relevant individuals in nFC-isPO. A total of 51 people participated, 35 (68.6%) completed the second survey in full, 11.8% (6 records) were missing. 24 (47.1%) of the participants in the second round did not partake in the first round. The structure of the participants covered a variety of occupational fields related to psycho-oncological care. Table 1 describes the characteristics of the participants.

**Table 1:** Participant characteristics

Characteristics of the participants from various fields of psycho-oncological care				
	**Round 1**		**Round 2**	
**Quality management touch points**	**N**	**%**	**N**	**%**
Yes	20	40.8%	35	68.6%
No	7	14.3%	11	21.6%
Missing	22	44.9%	5	9.8%
Total	49	100.0%	51	100.0%
**Working experience in years**	**N**		**N**	
Valid	27		46	
Missing	22		5	
Mean ± SD	13.22	± 11.01	12.24	± 11.19
Median	10		8	
Min	0		1	
Max	40		49	
Quartile				
1^st^	4		3	
2^nd^	10		8	
3^rd^	20		18.5	
**Professional role**	**N**	**%**	**N**	**%**
Project worker	7	18.4%	17	28.3%
Psychotherapist	6	15.8%	10	16.7%
Case manager	5	13.2%	4	6.7%
Physician in the Oncology Centre	4	10.5%	5	8.3%
Nurse practitioner	3	7.9%	3	5.0%
Network coordinator	3	7.9%	3	5.0%
Psychosocial specialist	3	7.9%	2	3.3%
Quality manager	1	2.6%	7	11.7%
Patient representative	1	2.6%	2	3.3%
isPO-onco-guide	1	2.6%	0	0.0%
Social worker	1	2.6%	0	0.0%
Physician in private practice	0	0.0%	0	0.0%
Other	2	5.3%	2	3.3%
Not reported	1	2.6%	5	8.3%
**Total**	**38**	**100.0%**	**60**	**100.0%**

### Consensus after round 1

Participants reached a strong consensus for 9 out of 88 (10.2%) indicators and a moderate consensus for another 20 (22.7%) indicators regarding relevance, i.e. the significance of the quality characteristic captured by the quality indicator for the care system. A total of 29 indicators were classified as relevant for the psycho-oncological care program. There was dissent for 58 (67.0%) indicators. No indicator was rejected. Data was missing for one indicator due to a technical error. 10 (11.36%) indicators were fully understandable and clear in definition to all survey participants. The remaining 78 (88.6%) indicators were rated comprehensive and clearly defined by at least 82.4% of the respondents. The results can be seen in Additional file 3.

### Results of the expert panels

In total, 28 indicators were added to the set by the authors while rechecking the indicators rated with dissent. In the second round, the members of the expert panel met in person under guidance of a moderator, discussed and evaluated the identified and the complemented indicators regarding data availability. A total of 57 indicators (29 strongly or moderately endorsed plus 28 additions) were evaluated. In 12 cases, implementation in health care practice was rejected due to lack of data availability regarding technical and legal aspects (e.g., data protection, lack of documentation, etc.). Subsequently, the preliminary set was adjusted to reflect care reality, several indicators were combined and the definitions were sharpened by the authors. 45 (79%) indicators were combined into 27. Five of these 27 were deferred as recommendations due to legal and technical uncertainties. The expert panel ended their work with 22 indicators to be implemented and tested in care practice.

### Implementation and piloting

The final set of 22 indicators has been operationalised in the information technology (IT)-supported documentation and assistance system (CAPSYS). CAPSYS was developed to record core data of patient care and contractual service provision, and to support the planning, management and monitoring of the pathway-guided and quality-assured patient care in the nFC-isPO [22]. In addition, a quality management module was developed within CAPSYS. Based on the documented data in CAPSYS, the quality indicators could be calculated and queried as a structured and standardised quality report. The quality report could be generated and retrieved internally for any selectable time period. Before the second round of the Delphi survey, the indicators included in the quality report were tested in practice for at least six months in four care networks in internal and cross-facility quality management.

### Consensus after round 2

In the second round, participants were asked to assess the 22 indicators in terms of their practicality. Consensus was reached for 16 (72.7%) indicators, thereof 6 (27.3%) with strong consensus and 10 (45.5%) with moderate consensus. There was dissent on 6 (27.3%) indicators. No indicator was rejected. Table 2 shows an overview of the results.

**Table 2:** Overview of results for the assessed quality indicators for Delphi round 2

Statistical results of the 2nd round of delphi assessing practical suitability													
		**Distribution**					**Measures of central tendency**				**Consensus**		
	**Short title**	**not relevant**	**rather not relevant**	**partly relevant**	**rather relevant**	**relevant**	**Mean ± SD**	**Median**	**Quartile**		**Direction**		**Level of consensus**
		(1)	(2)	(3)	(4)	(5)			1st	3rd	Consensus	Rejection	
**1**	Enrolments	2.9%	14.3%	17.1%	25.7%	40.0%	3.86 ± 1.192	4	3	5	65.7%	17.2%	dissent
**2**	Assignment to care level	25.7%	14.3%	2.9%	2.9%	54.3%	3.46 ± 1.804	5	1	5	57.2%	40.0%	dissent
**3**	isPO-onco-guide consultation	5.7%	5.7%	25.7%	25.7%	37.1%	3.83 ± 1.175	4	3	5	62.8%	11.4%	dissent
**4**	Withdrawals	2.9%	0.0%	20.0%	28.6%	48.6%	4.2 ± 0.964	4	4	5	77.2%	2.9%	moderate (+)
**5**	Reasons of withdrawals	8.6%	0.0%	8.6%	22.9%	60.0%	4.26 ± 1.197	5	4	5	82.9%	8.6%	moderate (+)
**6**	Report of critical events	2.9%	0.0%	2.9%	37.1%	57.1%	4.46 ± 0.817	5	4	5	94.2%	2.9%	strong (+ +)
**7**	Report of critical events—Involvement of additional caregivers	2.9%	0.0%	5.7%	42.9%	48.6%	4.34 ± 0.838	4	4	5	91.5%	2.9%	strong (+ +)
**8**	Number of initial services	2.9%	0.0%	5.7%	28.6%	62.9%	4.49 ± 0.853	5	4	5	91.5%	2.9%	strong (+ +)
**9**	Number of consultations	2.9%	0.0%	2.9%	14.3%	80.0%	4.69 ± 0.796	5	5	5	94.3%	2.9%	strong (+ +)
**10**	Average number of consultations per patient	2.9%	2.9%	5.7%	42.9%	45.7%	4.26 ± 0.919	4	4	5	88.6%	5.8%	moderate (+)
**11**	Average duration of consultations	5.7%	5.7%	5.7%	40.0%	42.9%	4.09 ± 1.121	4	4	5	82.9%	11.4%	moderate (+)
**12**	Time for organizing access	0.0%	2.9%	14.3%	22.9%	60.0%	4.4 ± 0.847	5	4	5	82.9%	2.9%	moderate (+)
**13**	Time to receive services	0.0%	0.0%	5.7%	22.9%	71.4%	4.66 ± 0.591	5	4	5	94.3%	0.0%	strong (+ +)
**14**	Time between consultations	0.0%	2.9%	20.0%	40.0%	37.1%	4.11 ± 0.832	4	4	5	77.1%	2.9%	moderate (+)
**15**	Time for organizing assessments	0.0%	8.6%	17.1%	28.6%	45.7%	4.11 ± 0.993	4	3	5	74.3%	8.6%	dissent
**16**	Time between service and documentation	2.9%	11.4%	25.7%	40.0%	20.0%	3.63 ± 1.031	4	3	4	60.0%	14.3%	dissent
**17**	Mean difference of HADS total scores	0.0%	0.0%	5.7%	25.7%	68.6%	4.63 ± 0.598	5	4	5	94.3%	0.0%	strong (+ +)
**18**	Percentage of improvements in anxiety and depression over time points T1 to T2	0.0%	2.9%	8.6%	14.3%	74.3%	4.6 ± 0.775	5	4	5	88.6%	2.9%	moderate (+)
**19**	Average reduction in anxiety and depression over time points T1 to T2	0.0%	2.9%	11.4%	17.1%	68.6%	4.51 ± 0.818	5	4	5	85.7%	2.9%	moderate (+)
**20**	Percentage of improvements in anxiety and depression over time points T1 to T3	0.0%	2.9%	11.4%	20.0%	65.7%	4.49 ± 0.818	5	4	5	85.7%	2.9%	moderate (+)
**21**	Average reduction in anxiety and depression over time points T1 to T3	2.9%	5.7%	5.7%	17.1%	68.6%	4.43 ± 1.037	5	4	5	85.7%	8.6%	moderate (+)
**22**	Patient satisfaction isPO-onco-guide consultation	8.6%	14.3%	14.3%	25.7%	37.1%	3.69 ± 1.345	4	3	5	62.8%	22.9%	dissent

**Threshold for consensus:** “strong consensus (+ +)” ≥ 90% in category 5 and 4; “moderate consensus (+)” ≥ 75% in category 5 and 4; “strong rejection (--)” ≥ 90% in category 2 and 1; “moderate rejection (-)” ≥ 75% in category 2 and 1; dissent: no unanimous group response

## Discussion

In this study a feasible and practical set of quality indicators was developed, operationalized in a quality report and pilot tested for a cross-sectoral psycho-oncological care program in the setting nFC-isPO. To date, few indicators related to cross-sectoral care of cancer patients have been integrated into the context of psycho-oncological routine care in Germany [11, 12]. The development of practice guidelines began internationally around 2008. In Germany, since around 2014, every institution has been obliged to develop and implement a written concept for psycho-oncological patient care in terms of a quality feature [10, 45–47]. Although there have been important milestones in the last decade, the road from evidence to implementation is still challenging [13, 26, 27, 48].

This research demonstrates the development, piloting, and finally definition of 16 trackable quality indicators. These 16 indicators reflect a relevant and comprehensive set covering psycho-oncology care across sectors, as well as Donabedian’s quality dimensions and numerous quality criteria according to JCAHO. A particular challenge was to overcome the sectoral boundaries in a shared set. In Germany, many cross-sectoral care programs are coordinated, e.g., through shared diagnostics or to save resources. This makes it difficult to apply quality indicators across sectors [49]. To avoid performance measurement for individual providers in the nFC-isPO and to ensure a holistic understanding, nFC-isPO quality indicators are always collected for an entire care network consisting of outpatient and inpatient providers. The nFC-isPO indicator set therefore emphasizes the psycho-oncological care program as a whole. Similar to Großimlinghaus [50], the indicator set consists of cross-sectoral and diagnosis-specific aspects. Despite the diagnosis-specific aspects, many of the indicators such as “average number of consultations per patient” or “time to receive services” could be transferred to other disease patterns with mentally distressed patients and similar organizational care structures. The set allows adaptations for different diagnoses, contextual differences, or even for different countries [50, 51].

Although no indicator was unanimously rejected, some aspects were perceived as significantly more irrelevant (rejection between 20 and 30%). In particular, indicators that go beyond the services provided by the nFC-isPO (e.g., “regular attendance of self-help group”, “number of relatives’ consultations”) and indicators related to documentation (e.g., “average time between data collections and documentation”) were rejected more strongly. One point of discussion on the expert panel was the relevance of the indicators for theoretical psycho-oncological care in general compared to the relevance for the concrete nFC-isPO. While some of the assessed quality demands were inherent in the structure of the nFC-isPO, it would be pointless to operationalize them in this setting. For example, “information availability for patients” would be unnecessary to record, as patient information is automatically given to the patient in the form of a supplementary sheet at enrolment in nFC-isPO. Nevertheless, it might generally be an important measure of the quality of psycho-oncology care. Another example was that the expert panel seemed to lean more towards emphasizing the relevance of the indicator “number of relative’s consultation” in the discussion, but voted only 55% in favour and 27% against (with a mean of 3.41 and standard deviation of 1.476). The wide dispersion suggests that the indicator might be relevant in general but not important for the nFC-isPO due to the structural organization. These aspects need to be considered, when revisiting and adjusting the set for other settings.

The results of this study contribute to national and international demands for improving psycho-oncological care structures. Defining and operationalizing psycho-oncological variables pursuing a uniform, cross-sectoral documentation goes far beyond the seven defined core variables of the first German evidence-based guideline on psycho-oncological diagnosis, counselling and treatment of adult cancer patients [12]. In this respect, the results support the goals of integrated and high-quality, psycho-oncological care [10, 11, 52]. The quality indicators developed can quantitatively cover the formulated goals of the NCP; the identification of psychosocial support needs as well as mental disorders in cancer patients and the provision of the necessary psycho-oncological care in inpatient and outpatient settings [11]. Particularly supportive measures for coping with the cancer (e.g., number of consultations, isPO-onco-guide counselling), relief of psychological and psychosomatic symptoms (e.g., mean difference of HADS total scores) as well as treatment adherence (e.g., reasons of withdrawal, time between services) are reflected in the set. In the medium term, there are considerations to supplement the set with indicators related to psycho-oncological care for relatives, quality of life and social reintegration. The feasibility of data collection and analysis was also tested area-wide as part of the nFC-isPO as required by the NCP [11]. By including inpatient and outpatient caregivers as well as cancer self-help groups (isPO-onco-guide), the set is cross-sectoral and might improve out-of-hospital psycho-oncological care by quantifying process and outcome quality (e.g., isPO-onco-guide consultations and patient satisfaction isPO-onco-guide consultations) [11].

Quality assurance through quality indicators can indirectly contribute to improving quality of care by making effects and outcomes visible [45, 53, 54]. As the lack of integration of indicators into information systems can be an immediate barrier in everyday use [55], this study aimed to link applicable quality indicators with standardized electronic documentation. Großimlinghaus et al. emphasize that the more use is made of existing, electronically available documents that can be extracted and evaluated with as little effort as possible, the better the feasibility of indicators [16, 55]. The strength of this research was that electronic implementation was part of the development process, i.e., evaluating data availability (phase 3) and testing validity in form of a quality report for at least six months (phase 4). Großimlinghaus et al. also emphasize that uniform data collection beyond the data already collected for billing purposes is essential for indicator projects. Therefore, the computerised documentation andassistance program (CAPSYS) [22] developed specifically for the nFC-isPO serves, among other things, as a standardized documentation system. Particularly with regard to numerous, cross-sectoral sites at which the nFC-isPO is carried out, standardized, consistent (electronic) documentation appears to be useful in order to record quality-relevant care data [16, 17]. The consented quality indicators were integrated into CAPSYS in the form of a quality report and enable quality comparisons [22]. By embedding the indicators digitally, the results can be accessed flexibly regardless of location and time. Thus, potentials for quality improvement can be quickly identified and used. The rapid transferability of quality assessments into practice and the linkage with quality improvement measures have been realized, which is important for a systematic approach to continuous quality improvement [56].

Team size was limited by the nature of the project, and there was inevitable turnover in the teams over the four years of the research. Participants were selected on the basis of their knowledge of the topic. Willingness to participate was assumed as all participants were project partners and already committed to the study. The clear inclusion criteria resulted in a relatively small pool of participants with high response rates, but low completion rates (55% and 68.6% respectively). Several studies have shown that the response rates for web surveys are much lower than for traditional surveys [34, 57, 58] and that the higher the number of items, the lower the completion rate [59]. This may explain why many experts abandoned the time-consuming web-based survey, especially in the first round. However, preliminary work on the size of expert panels has shown that a minimum of 20 participants is statistically relevant and can produce a valid expert opinion [60, 61]. In addition, recent studies have shown that small panels can produce reliable results and stable responses, especially when there are only a limited number of experts available in a field [62–64]. The high response rates of the small sized panel in this study are consistent with those observed in previous studies due to direct contact with participants [59].

Although consensus on the correct standard of methodological rigour is still lacking, the methodological changes may partially compromise the validity of this study [65]. The authors are aware that the specific sample of participants may threaten the external validity. Internal validity may be affected by the selection of the panel experts and the fact that the results are not necessarily replicable with comparable other groups [61]. In addition, the successive rounds of the survey resulted in ‘natural losses’ due to respondents dropping out. For pragmatic research reasons, dropouts and changes in the expert panel were inevitable as people left their jobs and the research project and/or new positions were filled. The professional heterogeneity of the panel is seen as a strength, as the participation of multi-perspective stakeholders is recommended and can increase the acceptability of quality indicators [34]. In contrast to a classical Delphi approach, only 41% of the participants in the second Delphi round were also present in the first round. Similar to the findings of Boulkedid et al. (2011), this may be equivalent to conducting distinct Delphi procedures, in which case it may be difficult to reach consensus.

Although the methodological design had to be modified due to the clinical practice setting, this study was developed and reported according to several guidelines and recommendations [34, 66–68]. Studies have shown that the selection of quality indicators based on consensus techniques is subject to great methodological variability [34, 69], and to date there is no 'one-size-fits-all' approach to identifying quality indicators for different settings. This study follows the methodological approach of the RAND/UCLA Appropriateness Method, combined with a modified consensus method, which is the first choice for identifying credible and valid indicators based on the opinion and experience of stakeholders with knowledge of the issue [34]. However, the further applicability and scientific evidence of the set of quality indicators should be demonstrated in subsequent studies to validate and update them in different care settings [61].

### Limitation

This study may have limitations. The indicator set was developed and applied specifically for a cross-sectoral psycho-oncological care program in the setting of nFC-isPO. These indicators proved feasible and appropriate for this purpose. With regard to the transferability of the indicator set to other settings, some adjustments certainly need to be made, but synergies are possible, especially for diseases with mental distress and cross-sectoral care approach [34, 51, 70]. Although no fixed reference ranges were defined in the beginning, initial empirical values for the indicators observed in everyday clinical practice could be determined. These values, in addition to evidence-based ones, can serve as an initial guide for setting a preliminary target range in the course of continuous revision of the set [16, 49]. Because of the SARS-COV-2 pandemic, direct patient involvement was not possible, but patient representatives were included. The research team tried to minimize the additional psychological burden and increased risk of infection for patients by reducing and postponing scheduled face-to-face interviews. The results of the patient interviews are still pending, but will be included into the set in the future [71]. Due to the small sample size and the low completion rate, this study lacks generalisability. Another limitation influencing panellists’ ratings is the level of evidence available for the indicators [55]. In the context of this study with potential indicators retrieving from very different sources, level of evidence was not presented to the participants from widely diverse work contexts to avoid bias. Although the lack of high level of evidence might reduce the generalizability of the findings [55], this is widespread in many health care settings and is the reason for using an expert panel methodology [72]. In addition, the lack of a gold standard for indicator development has been noted in several comparable studies [34, 73]. Counteracting this, the established RAM procedure provides a certain methodological quality by combining several systematic methods and concrete quality criteria [74]. This method presents indicators that are valid and described in sufficient detail so that their results are reproducible, comprehensive and classifiable. The development and use of indicators should be understood as a process, although an important milestone has been reached by creating a set of quality indicators for cross-sectoral, psycho-oncological care. Nevertheless, continuous further development is necessary [56].

## Conclusion

This study contributes to improving quality in cross-sectoral psycho-oncological care by providing a valid, comprehensive and feasible set of 16 quality indicators for cancer patients affected by mental disorders and emotional distress. Operationalizing the theoretical concept of quality into a set of quality indicators and integrating it into a standardized and digitized quality management system makes it possible to go beyond a purely descriptive presentation of performance. The practical test has shown that quality assurance and controlling based on a set covering cross-entity and entity-specific aspects of care is successful in this specific psycho-oncological setting. Further work is needed to continuously improve the set and check if these indicators can be transferred to similar settings.

## Supplementary Information

**Additional file 1.** 

**Additional file 2.**

**Additional file 3.**

## Data Availability

All data generated or analysed during this study are included in this published article and its supplementary files.

## Abbreviations

NCP: German National Cancer Plan
PDCA: Plan-Do-Check-Act cycle
nFC-isPO: New form of care integrated, cross-sectoral psycho-oncology
RAM: RAND/UCLA Appropriateness Method
JCAHO: Joint Commission on Accreditation of Healthcare Organizations
CIO: Centrum für Integrierte Onkologie
HADS: Hospital Anxiety and Depression Scale
