# Supplementary material for: Developing quality indicators for cross-sectoral psycho-oncology in Germany: combining the RAND/UCLA appropriateness method with a Delphi technique
Source: BMC Health Serv Res. 2023 Jun 8;23:599. doi: 10.1186/s12913-023-09604-3 (PMC10249931; doi:10.1186/s12913-023-09604-3)
Supplement: Supplementary file 1 — Additional file 1. [file 12913_2023_9604_MOESM1_ESM.pdf]

**Additional file 1.** Systematic literature review and search strategy.

| | |
|---|---|
| **Data bases** | PubMed, PsychINFO, Livivo, PSYNDEX, SpringerLink, Cochrane Library |
| **Period** | From year 2000 upwards when integrated care programs were implemented by the German statutory health insurance (SHI) as a new component of routine care to overcome sectoral barriers. |
| **Language** | English or German |
| **Keywords german** | (Qualitätsindikator [TiAB] OR Indikator [TiAB] OR Klinische Messgröße [TiAB] OR Qualitätsmessung [TiAB] OR Strukturqualität [TiAB] OR Prozessqualität [TiAB] OR Ergebnisqualität [TiAB]) AND (Psychoonkologisch [TiAB] OR Psychoonkologie [TiAB] OR Psychosozial [TiAB] OR Psychotherapie [TiAB] OR Psychotherapeutisch [TiAB] OR Depression [TiAB] OR Psychologisch [TiAB] OR Integrierte Versorgung [TiAB]) |
| **Keywords english** | (process indicator [TiAB] OR process measure [TiAB] OR process of care indicator [TiAB] OR process of care measure [TiAB] OR performance measure [TiAB] OR performance indicator [TiAB] OR Outcome indicator [TiAB] OR outcome measure [TiAB] OR benchmarking [TiAB] OR "benchmarking"[MeSH:NoExp]) AND (psycho-oncology [TiAB] OR psycho-oncological psychosocial OR depression [TiAB] OR "depression"[MeSH Terms] [TiAB] OR integrated care) |
| **Additionally** | Quality indicator databases, guidelines, institutions and project networks (see table 1) |

**Table 1.** Additional sources searched for indicators.

| **INSTITUTIONS** | | |
|---|---|---|
| AHRQ | Agency for Healthcare Research and Quality | USA |
| NQMC | National Quality Measures Clearinghouse | USA |
| FACT | Foundation for Accountability and Civic Trust | USA |
| ACHS | Australian Council on Healthcare Standards | Australia |
| NHS | National Health Service | UK |
| JCAHO | Joint Commission on Accreditation of Healthcare Organizations | USA |
| ASCO | American Society of Clinical Oncology | USA |
| WINHO | Wissenschaftliches Institut der Niedergelassenen Hämatologen und Onkologen | Germany |
| GKV QUINTH | Qualitätsindikatorenthesaurus | Germany |
| HSCIC | Health and Social Care Information Services | UK |
| WHO | World Health Organization | Switzerland |
| IQTIG | Institut für Qualitätssicherung und Transparenz im Gesundheitswesen | Germany |
| BQS | Bundesgeschäftstelle Qualitätssicherung | Germany |
| NICE | National Institute of Health and Clinical Excellence | UK |
| DAPO | Deutsche Arbeitsgemeinschaft für Psychosoziale Onkologie e.V. | Germany |
| IPOS | International Psycho-Oncology Society | USA |
| CAPO | Canadian Association of Psychosocial Oncology | Canada |
| APOS | American Psychosocial Oncology Society | USA |
| OEPPO | Austrian Platform of Psycho-Oncology | Austria |
| - | Oncosuisse | Switzerland |
| ICHOM | International Consortium for Health Outcomes Measurements | USA |

| | | |
|---|---|---|
| GAM | Indicator Registry Global AIDS Monitoring | USA |
| SWEDPOS | Swedish Psycho-Oncology Society | Sweden |
| SFPO | French Psycho-Oncology Society | France |
| RAND | RAND Corporation | USA |
| SEPO | Spanish Society of Psycho-Oncology | Spain |
| EPOS | epos Health Management | Germany |
| PONZ | Psycho-Social Oncology New Zealand | New Zealand |
| CMS | Centers for Medicare & Medicaid Services | USA |
| IOM | Institute of Medicine | USA |
| HBI | Health Benchmark Inc. | USA |
| NCCN | National Comprehensive Cancer Network | USA |
| NCI | National Cancer Institute | USA |
| NICQA | National Initiative for Cancer Care Quality | USA |
| NQF | National Quality Forum | USA |
| QMIS | Quality Measures Management Information System | USA |
| RAND QA Tools | Quality Assessment Tools from Rand Health | USA |

**QUALITY INDICATOR SETS**

| | | |
|---|---|---|
| AQUIK | Ambulante Qualitätsindikatoren und Kennzahlen | Germany |
| Schizophrenie | Weinmann, Stefan und Becker, Thomas: Qualitätsindikatoren für die integrierte Versorgung von Menschen mit Schizophrenie: Handbuch (2009) | Germany |
| QOPI | Quality Oncology Indicator Practice Initiative | USA |
| IQIP | International Quality Indicator Project - IQIP | USA |

**GUIDELINES**

| | | |
|---|---|---|
| S3-Leitlinie Psychoonkologie | S3-Leitlinie Psychoonkologische Diagnostik, Beratung und Behandlung von erwachsenen Krebspatienten (Registernummer 032 - 051OL) | Germany |
| NKP | Nationaler Krebsplan | Germany |
| NVL Unipolare Depression | S3-Leitlinie und Nationale Versorgungsleitlinie (NVL) Unipolare Depression (Registernummer nvl – 005) | Germany |

**PROJECT-INTERNAL CARE NETWORKS**

| | | |
|---|---|---|
| - | Uniklinik Köln | Germany |
| - | Maria-Hilf-Mönchengladbach | Germany |
| - | GFO Kliniken Troisdorf | Germany |
| - | Neuss St. Josef | Germany |
