# Supplementary material for: Developing quality indicators for cross-sectoral psycho-oncology in Germany: combining the RAND/UCLA appropriateness method with a Delphi technique
Source: BMC Health Serv Res. 2023 Jun 8;23:599. doi: 10.1186/s12913-023-09604-3 (PMC10249931; doi:10.1186/s12913-023-09604-3)
Supplement: Supplementary file 2 — Additional file 2. [file 12913_2023_9604_MOESM2_ESM.pdf]

**Additional file 2.** Findings literature review.

| # | Original indicator | Description | Resources | Author/Institution | Year | Country | Data Source |
|---|---|---|---|---|---|---|---|
| 1 | Adult's access to preventive/ambulatory health services: percentage of members 20 years an older who had an ambulatory or preventive care visit | This measure is used to assess the percentage of members 20 years and older who had an ambulatory or preventive care visit | National Quality Measures Clearingshouse (NQMC) | National Committee for Quality Assurance | Oct 15 | USA | Administrative clinical data |
| 2 | Assessment of integrated care: total score for the "Practice/Organization" characteristics on the Site Self Assessment (SSA) Evaluation Tool | This measure is used to assess the total score for the "Practice/Organization" characteristics on the Site Self Assessment (SSA) Evaluation Tool | National Quality Measures Clearingshouse (NQMC) | Maine Health Access Foundation | Jan 16 | Australia | Health professional survey |
| 3 | Continuing medical education, teaching, and research: does the hospital have duly updated basic protocols? | This measure is used to assess whether the hospital has duly updated basic protocols | National Quality Measures Clearingshouse (NQMC) | Spanish Society of Intensive and Critical Care and Units Coronary | Mar 11 | Spain | Organizational policies and procedures |
| 4 | Palliative care for adults: percentage of clinicians who receive education and training regarding palliative care concepts | This measure is used to assess the percentage of clinicians in the clinic who work with patients age 18 years and older who receive education and training regarding palliative care concepts. | National Quality Measures Clearingshouse (NQMC) | Institute for Clinical Systems Improvement | Nov 13 | USA | Health professional survey |
| 5 | Adult depression in primary care: percentage of patients who commit suicide at any time while managed in primary care | This measure is used to assess the percentage of patients age 18 years and older who commit suicide at any time while managed in primary care. | National Quality Measures Clearingshouse (NQMC) | Institute for Clinical Systems Improvement | Mar 16 | USA | Administrative clinical data; Electronic health/medical record |
| 6 | Depression care: percentage of patients 18 years of age or older with major depression or dysthymia who reached remission X months (+/- 30 days) after an index visit | This measure is used to assess the percentage of patients 18 years of age or older with major depression or dysthymia who reached remission 6 months (+/- 30 days) after an index visit | National Quality Measures Clearingshouse (NQMC) | MS Community Measurement | Jan 15 | USA | Administrative clinical data; Electronic health/medical record; paper medical record |

| | | | | | | | |
|---|---|---|---|---|---|---|---|
| 7 | Adult depression in primary care: percentage of patients who reached remission at X months (+/- 30 days) after diagnosis or initiating treatment, e.g., had a PHQ-9 score less than Y at X months (+/- 30 days) | This measure is used to assess the percentage of patients age 18 years and older who have reached remission at 12 months (+/- 30 days) after diagnosis or initiating treatment, e.g., had a Patient Health Questionnaire-9 (PHQ-9) score less than 5 at 12 months (+/- 30 days). | National Quality Measures Clearingshouse (NQMC) | Institute for Clinical Systems Improvement | Mar 16 | USA | Administrative clinical data; Electronic health/medical record |
| 8 | Adult depression in primary care: percentage of patients who responded to treatment at 12 months (+/- 30 days) after diagnosis or initiating treatment, e.g., had a PHQ score decreased by 50% from initial score at 12 months (+/- 30 days) | This measure is used to assess the percentage of patients age 18 years and older who responded to treatment at 12 months (+/- 30 days) after diagnosis or initiating treatment, e.g., had a Patient Health Questionnaire-9 (PHQ-9) score decreased by 50% from initial score at 12 months (+/- 30 days). | National Quality Measures Clearingshouse (NQMC) | Institute for Clinical Systems Improvement | Mar 16 | USA | Administrative clinical data; Electronic health/medical record |
| 9 | Helpfulness of counseling: mean score on six items asking about the helpfulness of counseling among young adults received counseling on selected topics | Helpfulness of counseling: mean score on six items asking about the helpfulness of counseling among young adults received counseling on selected topics | National Quality Measures Clearingshouse (NQMC) | - | - | - | - |
| 10 | HIV ambulatory care satisfaction: percentage of HIV positive adult patients who reported how often their mental health providers were responsible and professional | This measure is used to assess the percentage of HIV positive adult patients who reported how often their mental health providers were responsible and professional. | National Quality Measures Clearingshouse (NQMC) | New York State Department of Health AIDS Institute | Mar 02 | USA | Patient/ individual Survey |
| 11 | Hospital inpatients' experiences: percentage of adult inpatients who reported whether they would recommend this hospital to their friends and family | This measure is used to assess the percentage of adult inpatients who reported whether ("Definitely No," "Probably No," "Probably Yes," or "Definitely Yes") they were willing to recommend this hospital to their friends and family. | National Quality Measures Clearingshouse (NQMC) | Agency for Healthcare research and Quality; CAHPS Consortium, Centers for Medicare & Medicaid Services | Mar 17 | USA | Administrative clinical data; Patient/ individual survey |
| 12 | Behavioral health care patients' experiences: percentage of adult patients who reported whether they were provided information about treatment options | This composite measure indicates the percentage of adult patients who indicated ("Yes" or "No") whether they were provided information about treatment options. The "Information About Treatment Options" composite measure is based on two questions on the Experience of Care and Health Outcomes (ECHO) Survey. | National Quality Measures Clearingshouse (NQMC) | Agency for Healthcare Research and Quality, CAHPS Consortium, Harvard Medical School | Aug 04 | USA | Administrative clinical data; Patient/ individual survey |

| | | | | | | | |
|---|---|---|---|---|---|---|---|
| 13 | HIV ambulatory care satisfaction: percentage of HIV positive adolescent and adult patients who reported how often they could see their mental health providers soon enough for their needs when they needed an appointment | This measure is used to assess the percentage of HIV positive adult patients 18 years and older who reported how often they could see their mental health providers soon enough for their needs when they needed an appointment. | National Quality Measures Clearingshouse (NQMC) | New York State Department of Health AIDS Institute | Mar 02 | USA | Patient/ individual Survey |
| 14 | Health plan enrollees' satisfaction with care: adult health plan enrollees' overall rating of their health care | This measure is used to assess adult enrollees' overall rating of their health care. Enrollees rate all the health care they received on a scale from 0 to 10, where 0 is the worst health care possible and 10 is the best health care possible. | National Quality Measures Clearingshouse (NQMC) | Agency for Healthcare Research and Quality; CAHPS Consortium | May 12 | USA | Patient/ individual Survey |
| 15 | Behavioral health care patients' experiences: percentage of adult patients who reported how much help they received by the counseling or treatment they received | This single-item measure indicates the percentage of adult patients who reported how much help they received ("A lot," "Somewhat," "A little," or "Not at all") by the counseling or treatment they received. | National Quality Measures Clearingshouse (NQMC) | Agency for Healthcare research and Quality, CAHPS Consortium, Harvard Medical School | Aug 04 | USA | Administrative clinical data; Patient/ individual survey |
| 16 | Depression: percentage of patients diagnosed with depression with documented self-management goals set within the last 12 months | This population-based measure is used in primary care settings to assess the percentage of patients diagnosed with depression with documented self-management goals set within the last 12 months. Patient education and self-management skills have been important components of collaborative model interventions. | National Quality Measures Clearingshouse (NQMC) | HRSA health Disparities Collaboratives: Depression Collaborative | Jan 05 | USA | Registry Data |
| 17 | Cancer-depression and psychosocial distress: percentage of patients treated for depression for whom a response to therapy was documented within 6 weeks | This measure is used to assess the percentage of patients treated for depression for whom a response to therapy was documented within 6 weeks. | National Quality Measures Clearingshouse (NQMC) | RAND Corporation | Apr 10 | USA | Administrative clinical data; Paper medical record |
| 18 | Cancer-depression and psychosocial distress: percentage of patients diagnosed with depression for whom a treatment plan for depression was documented | This measure is used to assess the percentage of patients diagnosed with depression for whom a treatment plan for depression was documented. | National Quality Measures Clearingshouse (NQMC) | RAND Corporation | Apr 10 | USA | Administrative clinical data; Paper medical record |

| | | | | | | | |
|---|---|---|---|---|---|---|---|
| 19 | Informationsverfügbarkeit für Patienten bei Schizophrenie | Betroffene, die psychiatrische Dienste in Anspruch nehmen, sollten Informationen erhalten, die es ihnen ermöglichen, informierte Entscheidungen zu treffen. Die Voraussetzung hierfür ist, dass Patienten und Familienangehörige über Behandlungsmöglichkeiten, Erwartungen und Konsequenzen informiert werden. Um Patienten in therapeutische Prozesse einzubeziehen, müssen sie die notwendigen Informationen haben, um informierte Entscheidungen in Bezug auf ihre Versorgung treffen zu können. | GKV QUINTH - Qualitätsindikatoren Thesaurus | "Qualitätsindikatoren für die Integrierte Versorgung von Menschen mit Schizophrenie. Handbuch - gefördert vom AOK-Bundesverband (Weinmann/Becker) | Jan 10 | Germany | Combination of routine data and special survey |
| 20 | Verteilung der Diagnosen bei Schizophrenie | Den Anteil der in das Versorgungsmodell eingeschrieben Personen, die zu einem gegebenen Zeitpunkt<br>- die Diagnose einer Schizophrenie (F20),<br>- die Diagnose einer schizoaffektiven Störung (F25),<br>- die Diagnose einer sonstigen psychotischen Störung (andere F2-Subgruppen nach ICD-10) haben. | GKV QUINTH - Qualitätsindikatoren Thesaurus | "Qualitätsindikatoren für die Integrierte Versorgung von Menschen mit Schizophrenie. Handbuch - gefördert vom AOK-Bundesverband (Weinmann/Becker) | Jan 10 | Germany | Routine data (administrative data, that is available from a previous survey) |
| 21 | Einschreibungsrate | Den Anteil der Zielgruppe für die Integrierte Versorgung (alle Versicherten mit einer Diagnose der F2-Gruppe nach ICD-10), die in die IV eingeschrieben sind. Damit wird einerseits der Case-Mix der IV-Population abgebildet (B6a) und andererseits die Einschreibungsrate. Außerdem wird der Anteil der in der Bezugsregion Versicherten mit den oben genannten Diagnosen abgebildet, sodass eine Abschätzung möglich ist, wie viel Prozent der schizophren Erkrankten einer Region sich für das IV-Modell eingeschrieben haben. Mit diesem Indikator ist einerseits eine Abbildung des Case-Mix bezüglich der Zieldiagnosen möglich. Andererseits erfasst er die Einschreibungsrate. Wenn sich viele Personen mit einer Schizophrenie oder einer anderen psychotischen Störung in die IV einschreiben, kann dies ein Indikator für die Attraktivität des IV-Modells und eventuell auch für die wahrgenommene Qualität sein. Allerdings kann es viele Gründe geben, warum der Anteil der eingeschriebenen Personen in der Zielpopulation hoch oder niedrig ist. Dieser Indikator kann dann zu Rückfragen bezüglich der Attraktivität des Modells in der Region und zur Hinterfragung der Abläufe und Aufnahmeformalitäten führen. | GKV QUINTH - Qualitätsindikatoren Thesaurus | "Qualitätsindikatoren für die Integrierte Versorgung von Menschen mit Schizophrenie. Handbuch - gefördert vom AOK-Bundesverband (Weinmann/Becker) | Jan 10 | Germany | Routine data (administrative data, that is available from a previous survey) |

| | | | | | | | |
|---|---|---|---|---|---|---|---|
| 22 | Abbruch der Behandlung länger als 90 Tage bei Schizophrenie | Ein länger als 90 Tage andauernder Abbruch der Behandlung chronischpsychiatrisch Erkrankter kann für eine Beendigung der Therapie durch den Patienten sprechen, auf eine ungenügende Nachsorge hinweisen oder Folge einer Überweisung des Patienten in eine andere Einrichtung sein. Es kann sich aber auch um einen Patienten handeln, der keine Therapie mehr erhält, aber weiterhin bei einem Team eingeschrieben verbleibt, da er sich in Remission befindet und keine weitere Inanspruchnahme mehr notwendig ist. Eine frühzeitige Beendigung der Therapie (dazu gehören die ambulantenPatienten, die keine ambulante Nachsorge in Anspruch genommen haben, oder jene, die gegen ärztlichen Rat die Therapie beendet haben) ist weitverbreitet. In vielen Studien brachen 25 % der ambulanten Patienten die Therapie ab. In Studien konnte gezeigt werden, dass Personen, die ihre Behandlung beenden, häufig unzufrieden mit der Behandlung sind oder überzeugt sind, dass eine weitere Behandlung nicht notwendig sei. In einer anderen Untersuchung wurden Personen, die sich an eine empfohlene Therapie gehalten haben, mit denen verglichen, die frühzeitig ihre Behandlung abgebrochen haben. Dabei konnte festgestellt werden, dass eine frühzeitige Beendigung mit einem schlechteren Behandlungsergebnis und einem höheren Risiko der Rehospitalisierung verbunden ist (KILLAPSY et al.2000). Durch eine länger andauernde bessere Betreuung und Nachbetreuungvon Patienten, die ihre Therapie beenden, ist es möglich, Risikopatienten oder Schwachstellen im System zu erkennen. Klinische Interventionen, die einer frühzeitigen Beendigung der Therapie entgegenwirken sollen, zielen oft auf eine stärkere Einbeziehung der Vorstellungen des Betroffenen in die Therapie und eine aktive Kontaktaufnahme mit dem Betroffenen nach einem nicht wahrgenommenen Termin. | GKV QUINTH - Qualitätsindikatoren Thesaurus | "Qualitätsindikatoren für die Integrierte Versorgung von Menschen mit Schizophrenie. Handbuch - gefördert vom AOK-Bundesverband (Weinmann/Becker) | Jan 10 | Germany | Combination of routine data and special survey |

| | | | | | | | |
|---|---|---|---|---|---|---|---|
| 23 | Anteil der Patienten mit einer diagnostizierten Depression | Hier wird der Anteil der Patienten innerhalb der letzten 12 Monate beziffert, bei denen eine Depression diagnostiziert wurde. Depressionen zählen zu den häufigsten und gleichzeitig am meisten unterschätzten Erkrankungen. Ein Register der Patienten mit Depression erleichtert die nachhaltige Behandlung dieser Patientengruppe. Die Identifikation der Zielpopulation (Patienten mit Depression) in einem Register ist ferner die Voraussetzung für die Erhebung weiterer Indikatoren. | GKV QUINTH - Qualitätsindikatoren Thesaurus | Kassenärztliche Bundesvereinigung (KBV): KBV entwickelt Starter-Set ambulanter Qualitätsindikatoren. Ergebnisse des Projektes "AQUIK - Ambulante Qualitätsindikatoren und Kennzahlen" | Dez 09 | Germany | Special survey (special survey for the quality assurance) |
| 24 | Einschätzung Fallschwere bei Patienten mit Depression | Hier wird der Anteil der Patienten mit neu diagnostizierter Depression innerhalb der letzten 12 Monate beziffert, bei denen die Einschätzung der Fallschwere zu Beginn der Behandlung anhand eines validierten Hilfsmittels erfolgte (Validierte Hilfsmittel: z. B.: Patient Health Questionnaire-Depression (PHQ-D; Löwe et al., 2001; Spitzer et al., 1999), Beck-Depressionsinventar (BDI; Beck et al., 1961; Hautzinger, Bailer, Keller & Worrall, 1995; BDI II:Beck, Steer & Braun, 1996; Deutsch Hautzinger, Keller & Kühner, 2006), Hospital Anxiety and Depression Scale (HADS; Herrmann, Buss & Snaith, 1993), Klassifikation nach ICD-10-Kriterien). Der Schweregrad einer Depression ist ein zentrales Kriterium für Therapieentscheidungen und sollte daher anhand validierter Hilfsmittel möglichst objektiv erfasst werden. | GKV QUINTH - Qualitätsindikatoren Thesaurus | Kassenärztliche Bundesvereinigung (KBV): KBV entwickelt Starter-Set ambulanter Qualitätsindikatoren. Ergebnisse des Projektes "AQUIK - Ambulante Qualitätsindikatoren und Kennzahlen" | Dez 09 | Germany | Special survey (special survey for the quality assurance) |
| 25 | Beurteilung des Suizidrisikos bei Patienten mit Depression | Anteil der Patienten mit der Diagnose Depression innerhalb der letzten 12 Monate, deren Suizidrisiko bei jedem Arztbesuch beurteilt wurde. | GKV QUINTH - Qualitätsindikatoren Thesaurus | Kassenärztliche Bundesvereinigung (KBV): KBV entwickelt Starter-Set ambulanter Qualitätsindikatoren. Ergebnisse des Projektes "AQUIK - Ambulante Qualitätsindikatoren und Kennzahlen" | Dez 09 | Germany | Special survey (special survey for the quality assurance) |

| | | | | | | | |
|---|---|---|---|---|---|---|---|
| 26 | Anzahl der Suizide und Suizidversuche bei Schizophrenie | Etwa 10% der Menschen mit einer neu diagnostizierten Schizophrenie unternehmen innerhalb eines Jahres einen Suizidversuch, wobei Halluzinationen und vorheriges suizidales Verhalten die stärksten Risikofaktoren darstellten. Risikofaktoren, die sich als starke Prädiktorensuizidalen Verhaltens bei schizophrenen Patienten erwiesen haben und die in der Einschätzung der Suizidalität berücksichtigt werden sollten, sind: 1. Depressive Symptome; 2. Vorherige Suizidversuche; 3. Schwere der Erkrankung, insbesondere Halluzinationenund Denkstörungen; 4. Panikattacken und Angstsymptomatik; 5. Inadäquate neuroleptische Medikation; 6. Geringe Compliance; 7. Wiederholte kurze Krankenhausaufenthalte; 8. Hohe prämorbide Intelligenz und größere Einsicht in dieNatur der Erkrankung und ihre Konsequenzen; 9. Substanzmissbrauch; 10. Vorhandensein medikamenteninduzierter Akathisie; 11. Frühe Erkrankungsstadien; 12. Belastende Lebensereignisse; 13. Geringe soziale Unterstützung trotz besonderer Vorkehrungen und Schutzmaßnahmen kommt es dazu, dass Patienten, die an einer Schizophrenie leiden, sich selbst verletzen oder suizidieren. Zu den Selbstverletzungen gehört auch gesundheitsschädigendes Verhalten (gewollt oder ungewollt), welches spezifisch auf die psychiatrische Erkrankung zurückzuführen ist (ausgeschlossen sind hierbei Unfälle, wie beispielsweise Stürze). Die Inzidenz und der Outcome bei Selbstverletzung in stationärenpsychiatrischen Einrichtungen können mit den Betreuungs- und Überwachungspraktiken sowie mit der Ausbildung des Personals oder anderen Eigenschaften der Einrichtung assoziiert sein. Allerdings gibt es nur wenige Studien, die diesen Zusammenhang bisher untersucht haben. Die regelmäßige Überprüfung der Suizidalität des Betroffenen ist auch in der ambulanten Behandlung eine wichtige Aufgabe für psychiatrisch Tätige. | GKV QUINTH - Qualitätsindikatoren Thesaurus | "Qualitätsindikatoren für die Integrierte Versorgung von Menschen mit Schizophrenie. Handbuch - gefördert vom AOK-Bundesverband (Weinmann/Becker) | Jan 10 | Germany | Special survey (special survey for the quality assurance) |

| | | | | | | | |
|---|---|---|---|---|---|---|---|
| 27 | Regelmäßiger Besuch einer Selbsthilfegruppe bei Schizophrenie | Dieser Indikator erfasst den Anteil der eingeschriebenen Personen, die regelmäßig eine Selbsthilfegruppe besuchen. Selbsthilfegruppen sind mittlerweile ein wichtiger Bestandteil des Hilfesystems. In Selbsthilfegruppen findet ein Erfahrungsaustausch unter Betroffenen statt und Bewältigungsstrategien werden erarbeitet und weitergegeben. Auch psychoedukative Arbeit wird in Selbsthilfegruppen geleistet. Dies kann dazu führen, dass Krisen und Konflikte besser bewältigt werden und Ressourcen von Betroffenen aktiviert werden. Studien weisen darauf hin, dass Selbsthilfegruppen zur Verringerung der Rückfallrate beitragen können. Die Selbsthilfe spielt bei psychotischen Erkrankungen eine zunehmende Rolle. In vielen Regionen sind mittlerweile derartige Gruppen vorhanden. Betroffene mit einer Schizophrenie sollten auch von Therapeuten zum Besuch einer Selbsthilfegruppe ermutigt werden. Daher kann der Anteil der Personen, die regelmäßig eine Selbsthilfegruppe besuchen, auch ein Qualitätsindikator sein. | GKV QUINTH - Qualitätsindikatoren Thesaurus | "Qualitätsindikatoren für die Integrierte Versorgung von Menschen mit Schizophrenie. Handbuch - gefördert vom AOK-Bundesverband (Weinmann/Becker) | Jan 10 | Germany | Special survey (special survey for the quality assurance) |
| 28 | Demenz-Depressions-Screening | Anteil der Patienten ab 65 Jahren mit neu diagnostizierter Demenz innerhalb der letzten 12 Monate, die ein Screening auf das Vorliegen einer Depression innerhalb von drei Monaten nach Erstdiagnose der Demenz erhielten. | Ambulante Qualitätsindikatoren und Kennzahlen - AQUIK (KBV) | RAND Corporation 2007: RAND ACOVE-3-Set | 2009 | Germany | Routine data |
| 29 | Depression-Einschätzung Fallschwere | Anteil der Patienten mit neu diagnostizierter Depression innerhalb der letzten 12 Monate, bei denen die Einschätzung der Fallschwere spätestens zu Beginn der Behandlung anhand eines validierten Hilfsmittels erfolgte. | Ambulante Qualitätsindikatoren und Kennzahlen - AQUIK (KBV) | National Health Service (NHS) 2011: Quality and Outcomes Framework (QOP) | 2009 | Germany | Routine data |
| 30 | Praxismanagement-Weiterbildungsmaßnahmen | Alle ärztlichen und nicht-ärztlichen Mitglieder des Praxispersonals nahmen innerhalb der letzten 36 Monate nachweislich an Aus- bzw. Weiterbildungsmaßnahmen für Erste Hilfe teil | Ambulante Qualitätsindikatoren und Kennzahlen - AQUIK (KBV) | QEP-Qualitätsziel-Katalog kompakt, Version 2010, 1.3.4 (1); National Health Service (NHS) 2011: Quality and Outcomes Framework (QOF) | 2009 | Germany | - |

| | | | | | | | |
|---|---|---|---|---|---|---|---|
| 31 | Vorhandensein sektorenübergreifender (ambulant/stationärer) fachspezifischer Qualitätszirkel | Dieser Indikator erfasst das Vorhandensein regelmäßig (d.h. mindestens einmal im Quartal) stattfindender sektorenübergreifender und fachspezifischer Qualitätszirkel, an denen sowohl Klinikärzte als auch niedergelassene Ärzte und andere Therapeuten wie Pflegekräfte und Soziotherapeuten teilnehmen. Diese im Rahmen der Integrierten Versorgung stattfindenden Qualitätszirkel sind wichtige Instrumente des Qualitätsmanagements, können aber nur dann wirksam sein, wenn sie regelmäßig besucht werden, wenn sie zielorientiert sind, der Verbesserung der Behandlung und der Abläufe dienen und der Teilnehmer sie nicht lediglich als zusätzliche Fortbildung wahrnehmen. | - | "Qualitätsindikatoren für die Integrierte Versorgung von Menschen mit Schizophrenie. Handbuch - gefördert vom AOK-Bundesverband (Weinmann/Becker) | 2009 | Germany | Routine data within the IV |
| 32 | Psychotherapeutische Behandlung | Der Indikator erfasst den Anteil der Personen mit einer Schizophrenie, die im Rahmen ihres Therapieplans an einer problemorientierten Individual- oder Gruppenpsychotherapie teilnehmen. | - | "Qualitätsindikatoren für die Integrierte Versorgung von Menschen mit Schizophrenie. Handbuch - gefördert vom AOK-Bundesverband (Weinmann/Becker) | 2009 | Germany | GKV Routine data, Routine data within the IV |
| 33 | Zufriedenheit der Patienten mit der Behandlung | Der Indikator zeigt den Anteil der eingeschriebenen Personen an, die angeben, im Rahmen einer Patientenbefragung mit der Behandlung zufrieden zu sein. | - | "Qualitätsindikatoren für die Integrierte Versorgung von Menschen mit Schizophrenie. Handbuch - gefördert vom AOK-Bundesverband (Weinmann/Becker) | 2009 | Germany | Questionnaires on patient satisfaction |
| 34 | Drop-Out-Raten bei Behandlung der Ersterkrankung | Der Indikator erfasst den Anteil, der an einer Schizophrenie erstmalig Erkrankten, welche die Behandlung abgebrochen haben. | - | "Qualitätsindikatoren für die Integrierte Versorgung von Menschen mit Schizophrenie. Handbuch - gefördert vom AOK-Bundesverband (Weinmann/Becker) | 2009 | Germany | Routine documentation within the IV |

| | | | | | | | |
|---|---|---|---|---|---|---|---|
| 35 | Berufliche Wiedereingliederung | Der Indikator gibt den Anteil der Personen an, die sich für den Prozess der beruflichen Wiedereingliederung eignen und an diesem Teilnehmen. | - | "Qualitätsindikatoren für die Integrierte Versorgung von Menschen mit Schizophrenie. Handbuch - gefördert vom AOK-Bundesverband (Weinmann/Becker) | 2009 | Germany | Routine data within the IV/GKV |
| 36 | Patient/caregiver education | Numerator: Number of patients from the denominator who received patient education at least once during the measurement period regarding depression, depression treatment, prescribed medication and coping strategies Denominator: Number of adult patients with a diagnosis of major depression or dysthymia during the measurement period. | Petrosyan, Yelena; Sahakyan, Yeva; Barnsley, Jan M., Kuluski, Kerry; Liu, Barbara; Wodchis, Walter P. (2017): Quality indicators for care of depression in primary care settings: a systematic review. In: Systematic Reviews 6 (1), S. 126. DOI:10.1186/s13643-017-0530-7 | Shield T, Campdell S, Rogers A, Worrall A, Chew-Graham C, Gask L. Quality indicators for primary care mental health servies. Qual Saf Health Care. 2003;12(2):100-6 & 2003 & Canadian Mental Health Associataion (CMHA). Improving primary care mental health services. Results of a national quality care project. Published Feburary, 2012. https://cmha.bc.ca. Accessed 28 Oct 2014.& Worrall A, Rees A, Richards A, Rix S, Wright C, Lelliot P. The developmnet of a set of quality indicators to evaluate services for people with depression. J of Mental health. 2002;11(6):677-84 | 2017 | Canada | - |

| | | | | | | | |
|---|---|---|---|---|---|---|---|
| 37 | Access to care | Description: Patients are able to make a routine appointment to see a general practitioner within 2 days ('yes/no' response) | Petrosyan, Yelena; Sahakyan, Yeva; Barnsley, Jan M., Kuluski, Kerry; Liu, Barbara; Wodchis, Walter P. (2017): Quality indicators for care of depression in primary care settings: a systematic review. In: Systematic Reviews 6 (1), S. 126. DOI:10.1186/s13643-017-0530-7 | Shield T, Campdell S, Rogers A, Worrall A, Chew-Graham C, Gask L. Quality indicators for primary care mental health servies. Qual Saf Health Care. 2003;12(2):100-6 & Worrall A, Rees A, Richards A, Rix S, Wright C, Lelliot P. The developmnet of a set of quality indicators to evaluate services for people with depression. J of Mental health. 2002;11(6):677-84 | 2017 | Canada | - |
| 38 | Basisindikator B6: Verteilung der Diagnosen I | B6a: Diagnoseverteilung der Personen, die in einer Region in die Integrierte Versorgung (IV) eingeschrieben sind (Anteil der Personen mit Schizophrenie und anderen psychotischen Erkrankungen) | Kösters, Markus; Staudigl, Lena; Picca; Ann-Christien; Schmauß, Max; Becker, Thomas, Weinmann, Stefan (2017): Qualitätsindikatoren für die Behandlung von Menschen mit Schizophrenie - Ergebnisse einer Anwendungsstudie. In: Psychiatr Prax 44 (3), S. 163-171. Doi: 10.1055/s-0042-112300 | Qualitätsindikatoren für die Behandlung von Menschen mit Schizophrenie | 2017 | Germany | - |
| 39 | Basisindikator B6: Verteilung der Diagnosen II | Anteil der IV-Zielgruppe in einer Region, die tatsächlich in die IV eingeschrieben sind (Einschreibungsrate) | Kösters, Markus; Staudigl, Lena; Picca; Ann-Christien; Schmauß, Max; Becker, Thomas, Weinmann, Stefan (2017): Qualitätsindikatoren für die Behandlung von Menschen mit Schizophrenie - Ergebnisse einer Anwendungsstudie. In: | Qualitätsindikatoren für die Behandlung von Menschen mit Schizophrenie | 2017 | Germany | - |

| | | | | | | | |
|---|---|---|---|---|---|---|---|
| | | | Psychiatr Prax 44 (3), S. 163-171. Doi: 10.1055/s-0042-112300 | | | | |
| 40 | Basisindikator B8: Sektorenübergreifender Qualitätszirkel | Regelmäßig (mindestens einmal im Quartal) stattfindende sektorenübergreifende Qualitätszirkel, an denen Klinikärzte, niedergelassene Ärzte und andere IV-Therapeuten teilnehmen. | Kösters, Markus; Staudigl, Lena; Picca; Ann-Christien; Schmauß, Max; Becker, Thomas, Weinmann, Stefan (2017): Qualitätsindikatoren für die Behandlung von Menschen mit Schizophrenie - Ergebnisse einer Anwendungsstudie. In: Psychiatr Prax 44 (3), S. 163-171. Doi: 10.1055/s-0042-112300 | Qualitätsindikatoren für die Behandlung von Menschen mit Schizophrenie | 2017 | Germany | Information of the clinic management |
| 41 | Qualitätsindikator Q7: Abbruch der Behandlung länger als 90 Tage (Red-flag-Wert: >15%) | Anteil der Personen, die bei einem Arzt, in einer psychiatrischen Einrichtung oder einem niedergelassenen Arzt in Behandlung sind und in einem Zeitraum von 90 Tagen keine ärztliche psychiatrische Behandlung und keinen Kontakt mit einem Therapeuten im IV-System hatten. | Kösters, Markus; Staudigl, Lena; Picca; Ann-Christien; Schmauß, Max; Becker, Thomas, Weinmann, Stefan (2017): Qualitätsindikatoren für die Behandlung von Menschen mit Schizophrenie - Ergebnisse einer Anwendungsstudie. In: Psychiatr Prax 44 (3), S. 163-171. Doi: 10.1055/s-0042-112300 | Qualitätsindikatoren für die Behandlung von Menschen mit Schizophrenie | 2017 | Germany | - |
| 42 | Qualitätsindikator Q9: Case Manager (Red-flag-Wert: <60%) | Anteil der Personen, die in den letzten 6 Monaten Kontakt zu einem Case-Manager hatten. | Kösters, Markus; Staudigl, Lena; Picca; Ann-Christien; Schmauß, Max; Becker, Thomas, Weinmann, Stefan (2017): Qualitätsindikatoren für die Behandlung von Menschen mit Schizophrenie - Ergebnisse einer Anwendungsstudie. In: Psychiatr Prax 44 (3), S. 163- | Qualitätsindikatoren für die Behandlung von Menschen mit Schizophrenie | 2017 | Germany | - |

| | | | | | | | |
|---|---|---|---|---|---|---|---|
| | | | 171. Doi: 10.1055/s-0042-112300 | | | | |
| 43 | Qualitätsindikator Q12: Psychotherapeutische Behandlung | Anteil der Personen, die im Rahmen ihres Therapieplans an einer problemorientierten Individual- oder Gruppenpsychotherapie teilnehmen. | Kösters, Markus; Staudigl, Lena; Picca; Ann-Christien; Schmauß, Max; Becker, Thomas, Weinmann, Stefan (2017): Qualitätsindikatoren für die Behandlung von Menschen mit Schizophrenie - Ergebnisse einer Anwendungsstudie. In: Psychiatr Prax 44 (3), S. 163-171. Doi: 10.1055/s-0042-112300 | Qualitätsindikatoren für die Behandlung von Menschen mit Schizophrenie | 2017 | Germany | Survey |
| 44 | Qualitätsindikator Q15: Informationsverfügbarkeit für Patienten | Ausmaß der Verfügbarkeit adäquater Information zur Erkrankung und zur Behandlung des Betroffenen (aus Sicht des Patienten). | Kösters, Markus; Staudigl, Lena; Picca; Ann-Christien; Schmauß, Max; Becker, Thomas, Weinmann, Stefan (2017): Qualitätsindikatoren für die Behandlung von Menschen mit Schizophrenie - Ergebnisse einer Anwendungsstudie. In: Psychiatr Prax 44 (3), S. 163-171. Doi: 10.1055/s-0042-112300 | Qualitätsindikatoren für die Behandlung von Menschen mit Schizophrenie | 2017 | Germany | Survey |
| 45 | Patientenzufriedenheit mit der Behandlung | Anteil der Personen, die im Rahmen einer Patientenbefragung angeben, mit der Behandlung zufrieden zu sein. | Kösters, Markus; Staudigl, Lena; Picca; Ann-Christien; Schmauß, Max; Becker, Thomas, Weinmann, Stefan (2017): Qualitätsindikatoren für die Behandlung von Menschen mit Schizophrenie - Ergebnisse einer Anwendungsstudie. In: Psychiatr Prax 44 (3), S. 163-171. Doi: 10.1055/s-0042-112300 | Qualitätsindikatoren für die Behandlung von Menschen mit Schizophrenie | 2017 | Germany | Survey |

| | | | | | | | |
|---|---|---|---|---|---|---|---|
| 46 | Qualitätsindikator Q21: Berufliche Wiedereingliederung | Anteil der Personen, die sich für den Prozess der beruflichen Wiedereingliederung eignen und an diesem teilnehmen. | Kösters, Markus; Staudigl, Lena; Picca; Ann-Christien; Schmauß, Max; Becker, Thomas, Weinmann, Stefan (2017): Qualitätsindikatoren für die Behandlung von Menschen mit Schizophrenie - Ergebnisse einer Anwendungsstudie. In: Psychiatr Prax 44 (3), S. 163-171. Doi: 10.1055/s-0042-112300 & Großimlinghaus, I.; Hauth, I.; Falkai, P.; Janssen, B.; Deister, A.; Meyer-Lindenberg, A. et al. (2017): Aktuelle Empfehlungen der DGPPN für Schizophrenie-Qualitätsindikatoren. = DGPPN recommendations on quality indicators for schizophrenia. In: Der Nervenarzt 88 (7), S.779-786. DOI: 10.1007/s00115-017-0347-6 | Qualitätsindikatoren für die Behandlung von Menschen mit Schizophrenie; DGPPN-Qualitätsindikatoren | 2017 | Germany | Survey |
| 47 | Qualitätsindikator Q22: Anzahl der Suizide und Suizidversuche | Anzahl der vollzogenen Suizide und Anzahl der Suizidversuche/1000 Patienten. Aufteilung ambulant/stationär. | Kösters, Markus; Staudigl, Lena; Picca; Ann-Christien; Schmauß, Max; Becker, Thomas, Weinmann, Stefan (2017): Qualitätsindikatoren für die Behandlung von Menschen mit Schizophrenie - Ergebnisse einer Anwendungsstudie. In: Psychiatr Prax 44 (3), S. 163-171. Doi: 10.1055/s-0042-112300 & Großimlinghaus, I.; Hauth, I.; Falkai, P.; Janssen, B.; Deister, A.; Meyer-Lindenberg, A. et al. (2017): Aktuelle Empfehlungen der DGPPN für Schizophrenie-Qualitätsindikatoren. = DGPPN | Qualitätsindikatoren für die Behandlung von Menschen mit Schizophrenie; DGPPN-Qualitätsindikatoren | 2017 | Germany | Survey |

| | | | | | | | |
|---|---|---|---|---|---|---|---|
| | | | recommendations on quality indicators for schizophrenia. In: Der Nervenarzt 88 (7), S.779-786. DOI: 10.1007/s00115-017-0347-6 | | | | |
| 48 | Access: Waiting time start first treatment from first visit specialist <35 days | Access: Waiting time start first treatment from first visit specialist <35 days | Ouwens, M.; Hermens, R.; Hulscher, M.; Vonk-Okhuijsen, S.; Tja-Heijnen, V.; Termeer, R. et al. (2010): Development of indicators for patient-centered cancer care. In: Supportive Care in Cancer 18 (1), S.121-130. DOI:10.1007/s00520-009-0638-y | - | 2010 | Nether-lands | Questionnaire |
| 49 | Documentation of the current emotional well-being | Documentation of current emotional well-being was assessed within 1 month of the first visit with a medical oncologist | Jacobsen, P.B.; Shibata, D.; Siegel, E.M.; Lee, J.-H.; Alemany, C.A.; Brown, R. et al. (2009): Initial evaluation of quality indicators for psychosocial care of adults with cancer. In: Cancer Control 16 (4), S. 328-334. Doi:10.1171/107327480901600407 | - | 2009 | USA | Questionnaire |
| 50 | Indikator Behandlungsabbruch | Basisindikator | Hausen, A.; Glaeske, G. (2015): Die Bedeutung einer frühzeitigen begleitenden Evaluation für die Ableitung von Indikatoren zur Qualitätsmessung in der ambulanten psychiatrischen Integrierten Versorgung =The importance of an early accompanying evaluation of new care forms for the development of indicators for quality assurance in outpatient psychiatric integrated care. In: Gesundheitswesen 77 (5), S. | - | 2015 | Germany | Routine data |

| | | | | | | | |
|---|---|---|---|---|---|---|---|
| | | | 336-339. DOI: 10.1055/s-0034-1377031. | | | | |
| 51 | Indikator Einschreibungszahlen | Indikator mit einem Bezug zum Zusammenspiel der Vertragspartner. | Hausen, A.; Glaeske, G. (2015): Die Bedeutung einer frühzeitigen begleitenden Evaluation für die Ableitung von Indikatoren zur Qualitätsmessung in der ambulanten psychiatrischen Integrierten Versorgung =The importance of an early accompanying evaluation of new care forms for the development of indicators for quality assurance in outpatient psychiatric integrated care. In: Gesundheitswesen 77 (5), S. 336-339. DOI: 10.1055/s-0034-1377031. | - | 2015 | Germany | Routine data |
| 52 | Indikator Wartezeit bis zum Behandlungsbeginn | Indikator mit einem Bezug zur individuellen ambulanten Versorgungssituation . | Hausen, A.; Glaeske, G. (2015): Die Bedeutung einer frühzeitigen begleitenden Evaluation für die Ableitung von Indikatoren zur Qualitätsmessung in der ambulanten psychiatrischen Integrierten Versorgung=The importance of an early accompanying evaluation of new care forms for the development of indicators for quality assurance in outpatient psychiatric integrated care. In: Gesundheitswesen 77 (5), S. 336-339. DOI: 10.1055/s-0034-1377031. | - | 2015 | Germany | Routine data |

| | | | | | | | |
|---|---|---|---|---|---|---|---|
| 53 | Indikator Zugangswege | Indikator mit einem Bezug zum Zusammenspiel der Vertragspartner. | Hausen, A.; Glaeske, G. (2015): Die Bedeutung einer frühzeitigen begleitenden Evaluation für die Ableitung von Indikatoren zur Qualitätsmessung in der ambulanten psychiatrischen Integrierten Versorgung=The importance of an early accompanying evaluation of new care forms for the development of indicators for quality assurance in outpatient psychiatric integrated care. In: Gesundheitswesen 77 (5), S. 336-339. DOI: 10.1055/s-0034-1377031. | - | 2015 | Germany | Routine data |
| 54 | QI 4: Therapie/Einbeziehung von Selbsthilfe und Angehörigen | Z: Anzahl der Personen des Nenners, bei denen dokumentiert ist, dass sie Informationen zu einer Selbsthilfegruppe/ Angehörigengruppe erhalten hat N: Alle Patienten mit einer unipolaren depressiven Erkrankung in Behandlung innerhalb von 90 Tagen. | Großimlinghaus, I.; Falkai, P.; Gaebel, W.; Janssen, B.; Reich-Erkelenz, D.; Wobrock, T., Zielasek, J. (2013): Entwicklungsprozess der DGPPN-Qualitätsindikatoren. In: Der Nervenarzt 84 (3), S. 350-365. DOI: 10.1007/s00115-012-3705-4 | DGPPN-Qualitätsindikatoren Depression | 2013 | Germany | Account data, clinical documentation routine and KIS |
| 55 | Use of services for mental health reasons | At least one consultation for mental health reason in the past 12 months (family doctor/general practitioner, psychiatrist, other physicians, psychologist, nurse, social worker/ counselor/ psychotherapist, other health provider or professional) | Duhoux, Arnaud; Fournir, Louise; Gauvin, Lise; Roberge, Pasquale (2012): Quality of care for major depression and its determinants: A multilevel analysis. In: BMC Psychiatry 12. DOI: 10.1037/t03589-000 | - | 2012 | Canada | |
| 56 | Any form of psychotherapy or counseling | Help in form of psychotherapy or counseling in the past 12 months (among those with at least one consultation for mental health reason) | Duhoux, Arnaud; Fournir, Louise; Gauvin, Lise; Roberge, Pasquale (2012): Quality of care for major depression and its determinants: A multilevel | - | 2012 | Canada | |

| | | | | | | |
|---|---|---|---|---|---|---|
| | | | analysis. In: BMC Psychiatry 12. DOI: 10.1037/t03589-000 | | | |
| 57 | Adequate length session for psychotherapy or counseling | At least one session lasting 15 minutes or more of psychotherapy or counseling with one or other of the professionals consulted (among those who received help in the form of psychotherapy or counseling) | Duhoux, Arnaud; Fournir, Louise; Gauvin, Lise; Roberge, Pasquale (2012): Quality of care for major depression and its determinants: A multilevel analysis. In: BMC Psychiatry 12. DOI: 10.1037/t03589-000 | - | 2012 | Canada |
| 58 | Complete course of psychotherapy | 12 or more consultations for mental health reason in the past year. According to the Canadian recommendations, a minimum of 12 visits is required for a full course of psychotherapy (among those who received help in the form of psychotherapy or counseling) | Duhoux, Arnaud; Fournir, Louise; Gauvin, Lise; Roberge, Pasquale (2012): Quality of care for major depression and its determinants: A multilevel analysis. In: BMC Psychiatry 12. DOI: 10.1037/t03589-000 | - | 2012 | Canada |
| 59 | Antidepressant choice | Numerator: Number of patients from the denominator who were prescribed antidepressants using tertiary amine tricyclics, MAOIs (unless atypical depression is present), benzodiazepines or stimulants (except methylphenidate) as first- or second-line therapy Denominator: Number of adult patients with a diagnosis of major dysthymia during the measurement period ("negative indicator") | Petrosyan, Yelena; Sahakyan, Yeva; Barnsley, Jan M., Kuluski, Kerry; Liu, Barbara; Wodchis, Walter P. (2017): Quality indicators for care of depression in primary care settings: a systematic review. In: Systematic Reviews 6 (1), S. 126. DOI:10.1186/s13643-017-0530-7 | Nakajima, GA, Wenger, NS. Quality indicators for the care of depression in vulnerable elders. J Am Geriatr Soc. 2007;55 Suppl 2:S302-11 | 2017 | Canada |
| 60 | Depression remission at 6 month | Numerator: Number of patients from the denominator with an initial PHG-9 score greater than nine who achieve remission at 6 months as demonstrated by a 6-month (+- 30 days) PHQ-9 score of less than five. Denominator: Number of adult patients with a diagnosis of major depression or dysthymia and an initial PHQ-9 score greater than nine, during the measurement period. | Petrosyan, Yelena; Sahakyan, Yeva; Barnsley, Jan M., Kuluski, Kerry; Liu, Barbara; Wodchis, Walter P. (2017): Quality indicators for care of depression in primary care settings: a systematic review. In: Systematic Reviews 6 (1), S. 126. DOI:10.1186/s13643-017-0530-7 6 | National Quality measures Clearinghouse (NQMC) (NQF-endorsed) Adult depression in primary care | 2017 | Canada |

| | | | | | | | |
|---|---|---|---|---|---|---|---|
| 61 | Depression at 12 month | Numerator: Number of patients from the denominator with an initial PHG-9 score greater than nine who achieve remission at 12 months as demonstrated by a 12-month (+- 30 days) PHQ-9 score of less than five. Denominator: Number of adult patients with a diagnosis of major depression or dysthymia and an initial PHQ-9 score greater than nine, during the measurement period. | Petrosyan, Yelena; Sahakyan, Yeva; Barnsley, Jan M., Kuluski, Kerry; Liu, Barbara; Wodchis, Walter P. (2017): Quality indicators for care of depression in primary care settings: a systematic review. In: Systematic Reviews 6 (1), S. 126. DOI:10.1186/s13643-017-0530-7 | National Quality measures Clearinghouse (NQMC) (NQF-endorsed) Adult depression in primary care | 2017 | Canada | |
| 62 | Depression response at 6-month progress towards remission | Numerator: Number of patients from the denominator with an initial PHG-9 score greater than nine who achieve response at 6 months as demonstrated by a 6-month (+- 30 days) PHQ-9 score that is reduced by 50% or greater from the initial PHQ-9 score. Denominator: Number of adult patients with a diagnosis of major depression or dysthymia and an initial PHQ-9 score greater than nine, during the measurement period. | Petrosyan, Yelena; Sahakyan, Yeva; Barnsley, Jan M., Kuluski, Kerry; Liu, Barbara; Wodchis, Walter P. (2017): Quality indicators for care of depression in primary care settings: a systematic review. In: Systematic Reviews 6 (1), S. 126. DOI:10.1186/s13643-017-0530-7 | National Quality measures Clearinghouse (NQMC) (NQF-endorsed) Adult depression in primary care | 2017 | Canada | |
| 63 | Depression response at 12-month progress towards remission | Numerator: Number of patients from the denominator with an initial PHG-9 score greater than nine who achieve response at 12 months as demonstrated by a 12-month (+- 30 days) PHQ-9 score that is reduced by 50% or greater from the initial PHQ-9 score. Denominator: Number of adult patients with a diagnosis of major depression or dysthymia and an initial PHQ-9 score greater than nine, during the measurement period. | Petrosyan, Yelena; Sahakyan, Yeva; Barnsley, Jan M., Kuluski, Kerry; Liu, Barbara; Wodchis, Walter P. (2017): Quality indicators for care of depression in primary care settings: a systematic review. In: Systematic Reviews 6 (1), S. 126. DOI:10.1186/s13643-017-0530-7 | National Quality measures Clearinghouse (NQMC) (NQF-endorsed) Adult depression in primary care | 2017 | Canada | |
| 64 | QI 1: Diagnostik/Erfassung des Schweregerades/Schweregraderfassung | Z: Anzahl der Personen des Nenners, bei denen die Einschätzung der Fallschwere spätestens zu Beginn der Behandlung anhand der ICD- 10-Kriterien (F32-33) erfolgte N: Anzahl aller Personen mit neu diagnostizierter Depression oder erneuter depressiven Episode innerhalb eines Jahres. | Großimlinghaus, I.; Falkai, P.; Gaebel, W.; Janssen, B.; Reich-Erkelenz, D.; Wobrock, T., Zielasek, J. (2013): Entwicklungsprozess der DGPPN-Qualitätsindikatoren. In: Der Nervenarzt 84 (3), S. | DGPPN-Qualitätsindikatoren Depression | 2013 | Germany | Account data, clinical documentation routine and KIS |

| | | | | | | | |
|---|---|---|---|---|---|---|---|
| | | | 350-365. DOI: 10.1007/s00115-012-3705-4 | | | | |
| 65 | QI 3: Therapie allgemein/Aufklärung | Z: Anzahl der Personen des Nenners, bei denen dokumentiert ist, dass sie Informationen zu einer Selbsthilfegruppe/Angehörigengruppe erhalten haben N: Alle Patienten mit einer unipolaren depressiven Erkrankung in Behandlung innerhalb von 90 Tagen. | Großimlinghaus, I.; Falkai, P.; Gaebel, W.; Janssen, B.; Reich-Erkelenz, D.; Wobrock, T., Zielasek, J. (2013): Entwicklungsprozess der DGPPN-Qualitätsindikatoren. In: Der Nervenarzt 84 (3), S. 350-365. DOI: 10.1007/s00115-012-3705-5 | DGPPN-Qualitätsindikatoren Depression | 2013 | Germany | Account data, clinical documentation routine and KIS |
| 66 | QI 5: Therapie/Antidepressiva | Z: Anzahl der Personen des Nenners für die dokumentiert ist, dass ihnen eine medikamentöse Therapie mit einem Antidepressivum angeboten wurde N: Alle Personen mit akuter mittelgradiger depressiver Episode in Behandlung innerhalb von 90 Tagen. | Großimlinghaus, I.; Falkai, P.; Gaebel, W.; Janssen, B.; Reich-Erkelenz, D.; Wobrock, T., Zielasek, J. (2013): Entwicklungsprozess der DGPPN-Qualitätsindikatoren. In: Der Nervenarzt 84 (3), S. 350-365. DOI: 10.1007/s00115-012-3705-6 | DGPPN-Qualitätsindikatoren Depression | 2013 | Germany | Account data, clinical documentation routine and KIS |
| 67 | QI 6: Therapie/Kombination von Psycho- und Pharmakotherapie (Angebot) | Z: Zahl der Personen des Nenners, für die dokumentiert ist, dass ihnen eine Kombinationsbehandlung mit medikamentöser Therapie und Psychotherapie angeboten wurde N: Alle Personen mit akuten und chronischen schweren und rezidivierenden depressiven Episoden in Behandlung innerhalb von 90 Tagen. | Großimlinghaus, I.; Falkai, P.; Gaebel, W.; Janssen, B.; Reich-Erkelenz, D.; Wobrock, T., Zielasek, J. (2013): Entwicklungsprozess der DGPPN-Qualitätsindikatoren. In: Der Nervenarzt 84 (3), S. 350-365. DOI: 10.1007/s00115-012-3705-7 | DGPPN-Qualitätsindikatoren Depression | 2013 | Germany | Account data, clinical documentation routine and KIS |

| | | | | | | | |
|---|---|---|---|---|---|---|---|
| 68 | QI 7: Therapie/Kombination von Psycho- und Pharmakotherapie (Durchführung) | Z: Zahl der Personen des Nenners, die eine Kombinationsbehandlung mit medikamentöser Therapie und Psychotherapie erhalten haben. N: Alle Personen mit akuten und chronischen schweren und rezidivierenden depressiven Episoden in Behandlung, für die dokumentiert ist, dass ihnen eine Kombinationsbehandlung mit medikamentöser Therapie und Psychotherapie innerhalb von 90 Tagen angeboten wurde. | Großimlinghaus, I.; Falkai, P.; Gaebel, W.; Janssen, B.; Reich-Erkelenz, D.; Wobrock, T., Zielasek, J. (2013): Entwicklungsprozess der DGPPN-Qualitätsindikatoren. In: Der Nervenarzt 84 (3), S. 350-365. DOI: 10.1007/s00115-012-3705-8 | DGPPN-Qualitätsindikatoren Depression | 2013 | Germany | Account data, clinical documentation routine and KIS |
| 69 | QI 8: Therapie/ Angebot Psychotherapie | Z: Anzahl der Personen des Nenners, für die dokumentiert ist, dass ihnen eine Psychotherapie angeboten wurde N: Alle Personen mit leichter und mittelschwerer depressiver Episode in Behandlung innerhalb von 90 Tagen | Großimlinghaus, I.; Falkai, P.; Gaebel, W.; Janssen, B.; Reich-Erkelenz, D.; Wobrock, T., Zielasek, J. (2013): Entwicklungsprozess der DGPPN-Qualitätsindikatoren. In: Der Nervenarzt 84 (3), S. 350-365. DOI: 10.1007/s00115-012-3705-9 | DGPPN-Qualitätsindikatoren Depression | 2013 | Germany | Account data, clinical documentation routine and KIS |
| 70 | QI 9: Therapie/ Angebot und Durchführung einer Psychotherapie | Z: Anzahl der Personen des Nenners, die eine Psychotherapie erhalten haben. N: Alle Personen mit leichter bis mittelschwerer depressiver Episode in Behandlung innerhalb von 90 Tagen. | Großimlinghaus, I.; Falkai, P.; Gaebel, W.; Janssen, B.; Reich-Erkelenz, D.; Wobrock, T., Zielasek, J. (2013): Entwicklungsprozess der DGPPN-Qualitätsindikatoren. In: Der Nervenarzt 84 (3), S. 350-365. DOI: 10.1007/s00115-012-3705-10 | DGPPN-Qualitätsindikatoren Depression | 2013 | Germany | Account data, clinical documentation routine and KIS |
| 71 | QI 1 (sektorenübergreifend): Therapie/Behandlungskontinuität bei Suizidalität | Z: Anzahl der Personen des Nenners, für die dokumentiert ist, dass kurzfristig (maximal eine Woche nach Entlassung) eine Nachsorgeuntersuchung geplant wurde. N: Alle Personen mit stationärem Aufenthalt wergen Suizidalität innerhalb von 90 Tagen. | Großimlinghaus, I.; Falkai, P.; Gaebel, W.; Janssen, B.; Reich-Erkelenz, D.; Wobrock, T., Zielasek, J. (2013): Entwicklungsprozess der DGPPN-Qualitätsindikatoren. In: Der Nervenarzt 84 (3), S. 350-365. DOI: 10.1007/s00115-012-3705-11 | DGPPN-Qualitätsindikatoren Depression | 2013 | Germany | Account data, clinical documentation routine and KIS |

| | | | | | | | |
|---|---|---|---|---|---|---|---|
| 72 | QI 1: Strukturelle Voraussetzungen psychoonkologischer Versorgungsbereiche: Sektorenübergreifende Koordination der psychoonkologischen Versorgung | Z: Anzahl von Patienten, die Informationen über psychoonkologische Unterstützungsangebote erhalten haben N: Alle Krebspatienten mit Erstdiagnose, Rezidiv oder erster Fernmetastase Ziel: Die Einrichtung soll dem Patienten konkrete Ansprechpartner als Beispielreferenz benennen. Dadurch soll eine einrichtungsinterne- und übergreifende Netzwerkbildung gefördert werden. | Leitlinienprogramm Onkologie S3-Leitlinie Psychoonkologische Diagnostik, Beratung und Behandlung von erwachsenen Krebspatienten Version 1.1 - Januar 2014 AWMF-Registernummer: 0327051OL (Langversion) | Leitlinienprogramm Onkologie S3-Leitlinie Psychoonkologische Diagnostik, Beratung und Behandlung von erwachsenen Krebspatienten Version 1.1 - Januar 2014 AWMF-Registernummer: 0327051OL (Langversion) | 2014 | Germany | - |
| 73 | QI 2: Strukturelle Voraussetzungen psychoonkologischer Versorgungsbereiche: Selbsthilfegruppen | Z: Anzahl von Patienten, die Informationen über Unterstützungsangebote der Krebs-Selbsthilfegruppen/Krebsselbsthilfeorganisationen erhalten haben N: Alle Krebspatienten mit Erstdiagnose, Rezidiv oder erster Fernmetastase Anmerkung: Die Information kann per Flyer vermittelt werden, der Flyer sollte persönlich übergeben werden. Die betreffende Einrichtung gibt in dem Flyer konkret an, wo welches Angebot zu finden ist und nennt Ansprechpartner. | Leitlinienprogramm Onkologie S3-Leitlinie Psychoonkologische Diagnostik, Beratung und Behandlung von erwachsenen Krebspatienten Version 1.1 - Januar 2014 AWMF-Registernummer: 0327051OL (Langversion) | Leitlinienprogramm Onkologie S3-Leitlinie Psychoonkologische Diagnostik, Beratung und Behandlung von erwachsenen Krebspatienten Version 1.1 - Januar 2014 AWMF-Registernummer: 0327051OL (Langversion) | 2014 | Germany | - |
| 74 | QI 3 Diagnostik: Screening, Diagnostische Verfahren | Z: Anzahl von Patienten mit Einsatz von validierten und standardisierten Screening Instrumenten (z.B. das Distress-Thermometer oder die HADS-D) N: Alle Krebspatienten mit Erstdiagnose, Rezidiv oder erster Fernmetastase Anmerkung: Die Ablehnung eines Screenings sollte gesondert ausgewiesen werden. Falls kein Screening durchgeführt wurde, soll geschaut werden, ob ein diagnostisches Gespräch ggf. als Erstmaßnahme durchgeführt wurde. Wenn dies der Fall ist, wird dies als durchgeführtes Screening gewertet. | Leitlinienprogramm Onkologie S3-Leitlinie Psychoonkologische Diagnostik, Beratung und Behandlung von erwachsenen Krebspatienten Version 1.1 - Januar 2014 AWMF-Registernummer: 0327051OL (Langversion) | Leitlinienprogramm Onkologie S3-Leitlinie Psychoonkologische Diagnostik, Beratung und Behandlung von erwachsenen Krebspatienten Version 1.1 - Januar 2014 AWMF-Registernummer: 0327051OL (Langversion) | 2014 | Germany | - |

| | | | | | | | |
|---|---|---|---|---|---|---|---|
| 75 | QI 4 Diagnostik: Diagnostische Verfahren | Z: Anzahl von Patienten mit einem diagnostischen Gespräch zur Abklärung psychosozialer Belastungen und psychischer Komorbidität N: Alle Krebspatienten mit Erstdiagnose, Rezidiv oder erster Fernmetastase und mit positivem Screening auf psychosoziale Belastungen Definition "Diagnostisches Gespräch": Das diagnostische Gespräch beinhaltet die Identifikation von psychosozialen Belastungen, psychischen Störungen und weiteren Problemlagen mit dem Ziel der Beschreibung vorliegender Probleme und Störungen sowie deren Veränderung. Darüber hinaus erfolgt die Abklärung, ob diese Problemlagen subsyndromal sind oder die Kriterien für eine psychische Störung erfüllen. Die Abklärung und Zuordnung der vorliegenden Probleme und Störungen erfolgt entsprechend eines Klassifikationssystems (OCD-10 oder DSM IV), wobei bei der Diagnostik einer klinisch relevanten komorbiden Störung die Abgrenzung gegenüber somatischen Beschwerden oder einer angemessenen psychischen Reaktion auf die Tumorerkrankung sowie die zutreffende Berücksichtigung von biologisch-organischen Folgen der Krebserkrankungen bzw. Behandlung zu berücksichtigen sind. | Leitlinienprogramm Onkologie S3-Leitlinie Psychoonkologische Diagnostik, Beratung und Behandlung von erwachsenen Krebspatienten Version 1.1 - Januar 2014 AWMF-Registernummer: 0327051OL (Langversion) | Leitlinienprogramm Onkologie S3-Leitlinie Psychoonkologische Diagnostik, Beratung und Behandlung von erwachsenen Krebspatienten Version 1.1 - Januar 2014 AWMF-Registernummer: 0327051OL (Langversion) | 2014 | Germany | - |
| 76 | QI 5 Psychoonkologische Intervention: Konzepte und allgemeine Grundlagen für die Indikationsstellung psychoonkologischer Behandlungen | Z: Anzahl von Patienten mit Angebot einer psychotherapeutischen Einzel- und/oder Gruppenintervention N: Alle Krebspatienten mit Erstdiagnose, Rezidiv oder erster Fernmetastase und mit einer Angststörung (ICD- F43.2). | Leitlinienprogramm Onkologie S3-Leitlinie Psychoonkologische Diagnostik, Beratung und Behandlung von erwachsenen Krebspatienten Version 1.1 - Januar 2014 AWMF-Registernummer: 0327051OL (Langversion) | Leitlinienprogramm Onkologie S3-Leitlinie Psychoonkologische Diagnostik, Beratung und Behandlung von erwachsenen Krebspatienten Version 1.1 - Januar 2014 AWMF-Registernummer: 0327051OL (Langversion) | 2014 | Germany | - |
| 77 | QI 6 Psychoonkologische Interventionen: Konzepte und allgemeine Grundlagen für die Indikationsstellung psychoonkologischer Behandlung, psychosozialer Beratung | Z: Anzahl von Patienten mit Angebot einer psychosozialen Beratung N: Alle Krebspatienten mit Erstdiagnose, Rezidiv oder erster Fernmetastase Anmerkung: Eine psychosoziale Beratung soll durch Sozialarbeiter/Sozialpädagogen und Fachkräfte der Psychoonkologie persönlich angeboten werden (Vgl. persönliches Übergeben eines Flyers). | Leitlinienprogramm Onkologie S3-Leitlinie Psychoonkologische Diagnostik, Beratung und Behandlung von erwachsenen Krebspatienten Version 1.1 - Januar 2014 AWMF- | Leitlinienprogramm Onkologie S3-Leitlinie Psychoonkologische Diagnostik, Beratung und Behandlung von erwachsenen Krebspatienten Version 1.1 - Januar | 2014 | Germany | - |

| | | | | | | | |
|---|---|---|---|---|---|---|---|
| | | | Registernummer: 0327051OL (Langversion) | 2014 AWMF-Registernummer: 0327051OL (Langversion) | | | |
| 78 | QI 7 Patientenzentrierte Kommunikation: Fortbildungsmaßnahmen zur Verbesserung der kommunikativen Kompetenz der Behandler und deren Wirksamkeit | Z: Alle Ärztinnen/Ärzte und Pflegende mit Fort- und Weiterbildungsmaßnahmen zur Verbesserung ihrer kommunikativen Kompetenz N: Alle in der Onkologie tätigen Ärztinnen/Ärzte und Pflegende Anmerkung: Fortbildungs- und Weiterbildungsmaßnahmen zur Vermittlung spezifischer Gesprächskompetenzen: Postgradual; Anzahl von Unterrichtseinheiten muss nachgewiesen werden (z.B. Teilnehmerbescheinigung). Das Training sollte mindestens einen Umfang von 3 Tagen (24 Stunden) haben. | Leitlinienprogramm Onkologie S3-Leitlinie Psychoonkologische Diagnostik, Beratung und Behandlung von erwachsenen Krebspatienten Version 1.1 - Januar 2014 AWMF-Registernummer: 0327051OL (Langversion) | Leitlinienprogramm Onkologie S3-Leitlinie Psychoonkologische Diagnostik, Beratung und Behandlung von erwachsenen Krebspatienten Version 1.1 - Januar 2014 AWMF-Registernummer: 0327051OL (Langversion) | 2014 | Germany | - |
| 79 | Nr. 3 Suizidalitätsabklärung bei Patienten mit Depression | Werden alle Patienten mit Depression initial auf Suizidalität untersucht? | Schulz, Sven; Freytag, Antje; Chenot, Regine; Szecsenyi, Joachim. Qualitätsindikatoren für die Versorgung von Patienten mit Depression QISA | Szecsenyi, Joachim; Broge, Björn; Stock, Johannes. Qualitätsindikatorensystem für die ambulante Versorgung (QISA) | 2013 | Germany | Routine documentation in the electronic patient record |
| 80 | Nr. 4 Patientenaufklärung/-information zu Selbsthilfe- und Angehörigengruppen bei Patienten mit Depression | Werden die Patienten über ihre Erkrankung aufgeklärt und erhalten Informationen zu Selbsthilfegruppen bzw. Angehörigengruppen? | Schulz, Sven; Freytag, Antje; Chenot, Regine; Szecsenyi, Joachim. Qualitätsindikatoren für die Versorgung von Patienten mit Depression QISA | Szecsenyi, Joachim; Broge, Björn; Stock, Johannes. Qualitätsindikatorensystem für die ambulante Versorgung (QISA) | 2013 | Germany | Clinical documentation in the electronic patient record |

| | | | | | | | |
|---|---|---|---|---|---|---|---|
| 81 | Nr. 5 Patienten mit Depression, die eine antidepressive Pharmakotherapie erhalten | Wie viele Patienten mit Depression erhalten eine antidepressive Pharmakotherapie? | Schulz, Sven; Freytag, Antje; Chenot, Regine; Szecsenyi, Joachim. Qualitätsindikatoren für die Versorgung von Patienten mit Depression QISA | Szecsenyi, Joachim; Broge, Björn; Stock, Johannes. Qualitätsindikatorensystem für die ambulante Versorgung (QISA) | 2013 | Germany | Routine documentation in the electronic patient record, Account data of the payers |
| 82 | Nr. 6 Ausreichende Dauer der antidepressiven Pharmakotherapie nach Remission | Werden Patienten mit medikamentöser Behandlung ausreichend lange behandelt? | Schulz, Sven; Freytag, Antje; Chenot, Regine; Szecsenyi, Joachim. Qualitätsindikatoren für die Versorgung von Patienten mit Depression QISA | Szecsenyi, Joachim; Broge, Björn; Stock, Johannes. Qualitätsindikatorensystem für die ambulante Versorgung (QISA) | 2013 | Germany | Clinical documentation in the electronic patient record |
| 83 | Nr. 7 Patienten mit Depression, die eine Empfehlung zur psychotherapeutischen Behandlung erhalten | Wie viele Patienten mit Depression erhalten eine Empfehlung zur Psychotherapie? | Schulz, Sven; Freytag, Antje; Chenot, Regine; Szecsenyi, Joachim. Qualitätsindikatoren für die Versorgung von Patienten mit Depression QISA | Szecsenyi, Joachim; Broge, Björn; Stock, Johannes. Qualitätsindikatorensystem für die ambulante Versorgung (QISA) | 2013 | Germany | Routine documentation in the electronic patient record, Account data of the payers |
| 84 | Nr. 10 Verschreibung von Anxiolytika, Hypnotika bzw. Sedative bei Depression länger als vier Wochen | Ist die Verordnung von Axiolytika, Hypnotika bzw. Sedativa zeitlich begrenzt? | Schulz, Sven; Freytag, Antje; Chenot, Regine; Szecsenyi, Joachim. Qualitätsindikatoren für die Versorgung von Patienten mit Depression QISA | Szecsenyi, Joachim; Broge, Björn; Stock, Johannes. Qualitätsindikatorensystem für die ambulante Versorgung (QISA) | 2013 | Germany | Documentation in the electronic patient record |
| 85 | Indikator 1: Anteil der eingeschriebenen Versicherten in der Region | Wie groß ist der Anteil der eingeschriebenen Versicherten an allen potenziell einschreibfähigen Versicherten? (Attraktivität für Versicherte). | Broge, Björn; Stock, Johannes; Szecsenyi, Joachim. Allgemeine Indikatoren. Messgrößen für die Qualität regionaler Versorgungsmodelle. | Szecsenyi, Joachim; Broge, Björn; Stock, Johannes. Qualitätsindikatorensystem für die ambulante Versorgung (QISA) | 2009 | Germany | Insurance master data of the health insurance, Documentation of the registration |

| | | | | | | | |
|---|---|---|---|---|---|---|---|
| 86 | Indikator 2: Versichertenfluktuation | Der Indikator sagt aus, wie groß der Anteil der ausgetretenen Versicherten im Verhältnis zur Gesamtzahl der eingeschriebenen Versicherten des Vorjahres ist. (Attraktivität für Versicherte). | Broge, Björn; Stock, Johannes; Szecsenyi, Joachim. Allgemeine Indikatoren. Messgrößen für die Qualität regionaler Versorgungsmodelle. | Szecsenyi, Joachim; Broge, Björn; Stock, Johannes. Qualitätsindikatorensystem für die ambulante Versorgung (QISA) | 2009 | Germany | Documentation of the registrations and resignations and if applicable the reasons for resignation |
| 87 | Indikator 3: Anteil der eingeschriebenen Hausärzte in der Region | Der Indikator sagt aus, wie groß der Anteil der vertraglich in das Netz eingebundenn Hausärzte an allen Hausärzten der Region ist. (Attraktivität für Ärzte). | Broge, Björn; Stock, Johannes; Szecsenyi, Joachim. Allgemeine Indikatoren. Messgrößen für die Qualität regionaler Versorgungsmodelle. | Szecsenyi, Joachim; Broge, Björn; Stock, Johannes. Qualitätsindikatorensystem für die ambulante Versorgung (QISA) | 2009 | Germany | KV-Data or GKV-Routine data, Documentation of registration |
| 88 | Indikator 4: Arztfluktuation | Der Indikator sagt aus, wie groß der Anteil der Arztaustritte im Bezugszeitraum im Verhältnis zur Gesamtzahl der teilnehmenden Hausärzte ist. (Attraktivität für Ärzte). | Broge, Björn; Stock, Johannes; Szecsenyi, Joachim. Allgemeine Indikatoren. Messgrößen für die Qualität regionaler Versorgungsmodelle. | Szecsenyi, Joachim; Broge, Björn; Stock, Johannes. Qualitätsindikatorensystem für die ambulante Versorgung (QISA) | 2009 | Germany | Registration documents of the network |
| 89 | Indikator 6: Steuerung und Unterstützung der Qualitätszirkelarbeit | Der Indikator sagt aus, wie systematisch und mit welcher Intensität die Qualitätszirkelarbeit im Versorgungsmodell aufgebaut und untereinander abgestimmt ist. Nur wenn beispielsweise ein Netzmanagement über wesentliche Informationen zu den stattfindenden Qualitätszirkeln verfügt, kann es diese Informationen zielgerichtet im Hinblick auf die regionalen Versorgungsziele verwenden und die Qualitätszirkelarbeit unterstützen. Dies lässt sich nur mit Blick auf verschiedene Aspekte bewerten. Der Indikator misst, wie viele dieser Aspekte in einem Versorgungsmodell erfüllt sind. (Qualitätszirkelarbeit). | Broge, Björn; Stock, Johannes; Szecsenyi, Joachim. Allgemeine Indikatoren. Messgrößen für die Qualität regionaler Versorgungsmodelle. | Szecsenyi, Joachim; Broge, Björn; Stock, Johannes. Qualitätsindikatorensystem für die ambulante Versorgung (QISA) | 2009 | Germany | Information of the management, Evaluation sheet |
| 90 | Indikator 7: Anteil der Ärzte mit Teilnahme an mindestens vier Qualitätszirkeln/Jahr | Der Indikator sagt aus, wie groß der Anteil der Netzärzte ist, die im vergangenen Jahr an mindestens vier Qualitätstreffen teilgenommen haben, gemessen an allen Netzärzten. (Qualitätszirkelarbeit) | Broge, Björn; Stock, Johannes; Szecsenyi, Joachim. Allgemeine Indikatoren. Messgrößen für die Qualität regionaler Versorgungsmodelle. | Szecsenyi, Joachim; Broge, Björn; Stock, Johannes. Qualitätsindikatorensystem für die | 2009 | Germany | Documentation of the quality circle work |

| | | | | | | | |
|---|---|---|---|---|---|---|---|
| | | | | ambulante Versorgung (QISA) | | | |
| 91 | Indikator 9: Qualität und Verfügbarkeit von Informationen zum Versorgungsmodell und zu krankheitsspezifischen Themen für Versicherte | Der Indikator stellt das Spektrum der Maßnahmen dar, die der Information der Versicherten dienen. Relevante Aspekte zur Qualität und Verfügbarkeit solcher Materialen in einem Netz enthält Tab. 3. (Information der Versicherten). | Broge, Björn; Stock, Johannes; Szecsenyi, Joachim. Allgemeine Indikatoren. Messgrößen für die Qualität regionaler Versorgungsmodelle. | Szecsenyi, Joachim; Broge, Björn; Stock, Johannes. Qualitätsindikatorensystem für die ambulante Versorgung (QISA) | 2009 | Germany | Evaluation sheet with the consensus of the network |
| 92 | Indikator 10: Anteil der Ärzte, die an der Arztbefragung teilgenommen haben | Der Indikator gibt den Anteil der Netzärzte an, die an der Arztbefragung teilgenommen haben, gemessen an allen Netzteilnehmern (Rücklaufquote). (Kommunikation im Versorgungsmodell). | Broge, Björn; Stock, Johannes; Szecsenyi, Joachim. Allgemeine Indikatoren. Messgrößen für die Qualität regionaler Versorgungsmodelle. | Szecsenyi, Joachim; Broge, Björn; Stock, Johannes. Qualitätsindikatoren-system für die ambulante Versorgung (QISA) | 2009 | Germany | Response rate of the physicians survey |
| 93 | Indikator 12: Anteil der Ärzte, die sich über Ziele und Aktivitäten im Versorgungsmodell gut informiert fühlen | Der Indikator gibt einen Hinweis darauf, wie gut sich die Mitglieder über Ziele und Aktivitäten im regionalen Versorgungsmodell informiert fühlen. (Kommunikation im Versorgungsmodell). | Broge, Björn; Stock, Johannes; Szecsenyi, Joachim. Allgemeine Indikatoren. Messgrößen für die Qualität regionaler Versorgungsmodelle. | Szecsenyi, Joachim; Broge, Björn; Stock, Johannes. Qualitätsindikatoren-system für die ambulante Versorgung (QISA) | 2009 | Germany | Physicians Survey |
| 94 | Indikator 13: Anteil der zufriedenen Versicherten im Netz | Der Indikator gibt den Anteil der Versicherten im Netz an, die mit der Versorgung im Netz insgesamt zufrieden sind (Zufriedenheit der Versicherten). | Broge, Björn; Stock, Johannes; Szecsenyi, Joachim. Allgemeine Indikatoren. Messgrößen für die Qualität regionaler Versorgungsmodelle. | Szecsenyi, Joachim; Broge, Björn; Stock, Johannes. Qualitätsindikatorensystem für die ambulante Versorgung (QISA) | 2009 | Germany | Survey of insured people |
| 95 | Indikator 14: Anteil der Versicherten, die das Versorgungsmodell weiterempfehlen würden | Der Indikator gibt einen Hinweis darauf, wie groß der Anteil der Versicherten ist, die so zufrieden mit der Betreuung im Netz sind, dass sie es anderen weiterempfehlen würden. (Zufriedenheit der Versicherten). | Broge, Björn; Stock, Johannes; Szecsenyi, Joachim. Allgemeine Indikatoren. Messgrößen für die Qualität regionaler Versorgungsmodelle. | Szecsenyi, Joachim; Broge, Björn; Stock, Johannes. Qualitätsindikatorensystem für die ambulante Versorgung (QISA) | 2009 | Germany | Survey of insured people |

| | | | | | | | |
|---|---|---|---|---|---|---|---|
| 96 | Indikator 15: Anteil der Ärzte, die mit den Ergebnissen des Versorgungsmodells im vergangenen Jahr zufrieden sind. | Der Indikator gibt einen Hinweis darauf, wie hoch der Anteil der mit den Netzergebnissen zufriedenen Netzärzte ist. (Zufriedenheit der Ärzte). | Broge, Björn; Stock, Johannes; Szecsenyi, Joachim. Allgemeine Indikatoren. Messgrößen für die Qualität regionaler Versorgungsmodelle. | Szecsenyi, Joachim; Broge, Björn; Stock, Johannes. Qualitätsindikatorensystem für die ambulante Versorgung (QISA) | 2009 | Germany | Physician survey |
| 97 | Indikator 20: Anteil der Patienten mit Einhaltung der vereinbarten Wartezeit | Der Indikator gibt einen Hinweis darauf, wie groß der Anteil der eingeschriebenen Versicherten ist, die bei geplanten Arztterminen nicht über die im Netzwerk vereinbarten, maximalen Wartezeiten hinaus warten mussten. (Erreichbarkeit und Wartezeiten). | Broge, Björn; Stock, Johannes; Szecsenyi, Joachim. Allgemeine Indikatoren. Messgrößen für die Qualität regionaler Versorgungsmodelle. | Szecsenyi, Joachim; Broge, Björn; Stock, Johannes. Qualitätsindikatorensystem für die ambulante Versorgung (QISA) | 2009 | Germany | Survey of insured people |
| 98 | Indikator 21: Beurteilung der Erreichbarkeit der Ärzte aus Sicht der eingeschriebenen Versicherten | Der Indikator gibt Hinweise darauf, inwieweit die telefonische Erreichbarkeit, die Öffnungszeiten und die Möglichkeit, passende Termine zu erhalten, aus Sicht der Versicherten zufriedenstellend sind. (Erreichbarkeit und Wartezeiten). | Broge, Björn; Stock, Johannes; Szecsenyi, Joachim. Allgemeine Indikatoren. Messgrößen für die Qualität regionaler Versorgungsmodelle | Szecsenyi, Joachim; Broge, Björn; Stock, Johannes. Qualitätsindikatorensystem für die ambulante Versorgung (QISA) | 2009 | Germany | Survey of insured people |
| 99 | Indikator 28: Zahl der stationären Notfallaufnahmen je 1000 eingeschriebene Versicherten | Der Indikator gibt an, wie viele stationäre Notfallaufnahmen von eingeschriebenen Versicherten im Bezugszeitraum stattgefunden haben. (Patientensteuerung). | Broge, Björn; Stock, Johannes; Szecsenyi, Joachim. Allgemeine Indikatoren. Messgrößen für die Qualität regionaler Versorgungsmodelle | Szecsenyi, Joachim; Broge, Björn; Stock, Johannes. Qualitätsindikatorensystem für die ambulante Versorgung (QISA) | 2009 | Germany | GKV Routine data |
| 100 | Anteil der psychoonkologischen Gespräche | Wie viele onkologische Patienten nehmen mindestens ein psychoonkologisches Gespräch in Anspruch? | Project internal care network | - | - | - | - |
| 101 | Anzahl der psychoonkologischen Gespräche | Wie viele psychoonkologische Gespräche hat der onkologische Patient in Anspruch genommen? | Project internal care network | - | - | - | - |
| 102 | Gesprächsdauer | Wie lange dauern die psychoonkologischen Gespräche im Schnitt? | Project internal care network | - | - | - | - |
| 103 | Vollständige Dokumentation | Anteil aller vollständig dokumentierten Fälle. | Project internal care network | - | - | - | - |

| | | | | | | | |
|---|---|---|---|---|---|---|---|
| 104 | Anzahl der PSO-Primärfall-Gespräche | Anzahl der PSO-Primärfall- Gespräche. | Project internal care network | - | - | - | - |
| 105 | Anzahl der PSO Rezidivfall-Gespräche | Anzahl der PSO Rezidivfall- Gespräche. | Project internal care network | - | - | - | - |
| 106 | Anzahl der PSO-Verlaufsgespräche | Anzahl der PSO-Verlaufsgespräche. | Project internal care network | - | - | - | - |
| 107 | Anzahl der PSO-Nachsorge Gespräche | Anzahl der PSO-Nachsorge Gespräche. | Project internal care network | - | - | - | - |
| 108 | Anzahl der PSO-Angehörigengespräche | Anzahl der PSO-Angehörigengespräche. | Project internal care network | - | - | - | - |
| 109 | Anzahl der psychosozialen Interventionen | Anzahl der psychosozialen Interventionen. | Project internal care network | - | - | - | - |
