# Supplementary material for: Developing quality indicators for cross-sectoral psycho-oncology in Germany: combining the RAND/UCLA appropriateness method with a Delphi technique
Source: BMC Health Serv Res. 2023 Jun 8;23:599. doi: 10.1186/s12913-023-09604-3 (PMC10249931; doi:10.1186/s12913-023-09604-3)
Supplement: Supplementary file 3 — Additional file 3. [file 12913_2023_9604_MOESM3_ESM.pdf]

Additional file 3. Overview of results for the assessed quality indicators for round 1.

	STATISTICAL RESULT 1st ROUND OF DELPHI ASSESSING RELEVANCE AND COMPREHENSIBILTY															
		RELEVANCE													COMPREHEN-SIBILITY	
		Distribution					Measures of central tendency					Consensus				
	**Short title**	**not relevant** (1)	**rather not relevant** (2)	**partly relevant** (3)	**rather relevant** (4)	**relevant** (5)	**Mean ± SD**		**Median**	**Quartile** 1st	3rd	**Direction** Consensus	Rejection	**Level of consensus**	**N**	**%**
1a	Number of enrolled patients	0.0%	2.9%	17.6%	17.6%	61.8%	4.38	±0.888	5	4	5	79.4%	2.9%	moderate (+)	32	94.1%
1b	Percentage of enrolled patients	0.0%	2.9%	8.8%	20.6%	67.6%	4.53	±0.788	5	4	5	88.2%	2.9%	moderate (+)	33	97.1%
2a	Access via Oncology Center	5.9%	5.9%	26.5%	17.6%	44.1%	3.88	±1.225	4	3	5	61.7%	11.8%	dissent	32	94.1%
2b	Access via outpatient caregivers	5.9%	5.9%	23.5%	23.5%	41.2%	3.88	±1.2	4	3	5	64.7%	11.8%	dissent	32	94.1%
3a	Participation according to medical recommendation	0.0%	5.9%	41.2%	8.8%	44.1%	3.91	±1.055	4	3	5	52.9%	5.9%	dissent	33	97.1%
3b	Refusal of participation after medical recommendation	0.0%	2.9%	26.5%	23.5%	47.1%	4.15	±0.925	4	3	5	70.6%	2.9%	dissent	33	97.1%
4a	Patient satisfaction	0.0%	0.0%	5.9%	26.5%	67.6%	4.62	±0.604	5	4	5	94.1%	0.0%	strong (++)	33	97.1%
4b	Patient dissatisfaction	0.0%	2.9%	5.9%	20.6%	70.6%	4.59	±0.743	5	4	5	91.2%	2.9%	strong (++)	33	97.1%
5	Patient-Caregiver relationship	0.0%	0.0%	14.7%	20.6%	64.7%	4.5	±0.749	5	4	5	85.3%	0.0%	moderate (+)	28	82.4%
6	Coping with psychological and psychosocial problems	0.0%	2.9%	17.6%	2.9%	76.5%	4.53	±0.896	5	4.75	5	79.4%	2.9%	moderate (+)	30	88.2%
7	Patient recommendation	0.0%	2.9%	17.6%	29.4%	50.0%	4.26	±0.864	4.5	4	5	79.4%	2.9%	moderate (+)	33	97.1%
8	isPO-onco-guide consultation	0.0%	2.9%	20.6%	29.4%	47.1%	4.21	±0.88	4	3.75	5	76.5%	2.9%	moderate (+)	33	97.1%
9	Availability of service provider	5.9%	5.9%	14.7%	38.2%	35.3%	3.91	±1.138	4	3	5	73.5%	11.8%	dissent	33	97.1%
10	Professional reintegration	0.0%	23.5%	32.4%	23.5%	20.6%	3.41	±1.076	3	2.75	4	44.1%	23.5%	dissent	28	82.4%
11	Information availability for patients	2.9%	5.9%	20.6%	17.6%	52.9%	4.12	±1.122	5	3	5	70.5%	8.8%	dissent	31	91.2%
12	Patient information on self-help and family groups	0.0%	8.8%	23.5%	32.4%	35.3%	3.94	±0.983	4	3	5	67.7%	8.8%	dissent	32	94.1%
13	Patient information on support services	2.9%	11.8%	11.8%	41.2%	32.4%	3.88	±1.094	4	3	5	73.6%	14.7%	dissent	31	91.2%

14	Average number of case manager consultations	0.0%	5.9%	38.2%	20.6%	35.3%	3.85	±0.989	4	3	5	55.9%	5.9%	dissent	33	97.1%
15	Regular attendance of a self-help group	8.8%	20.6%	41.2%	17.6%	11.8%	3.03	±1.114	3	2	4	29.4%	29.4%	dissent	32	94.1%
16a	Number of physicians participating in the care program	0.0%	12.9%	29.0%	6.5%	51.6%	3.97	±1.169	5	3	5	58.1%	12.9%	dissent	30	96.8%
16b	Percentage of physicians participating in the care program	3.2%	19.4%	22.6%	19.4%	35.5%	3.65	±1.253	4	3	5	54.9%	22.6%	dissent	29	93.5%
16c	Number of Oncology centers participating in the care program	3.2%	12.9%	22.6%	6.5%	54.8%	3.97	±1.278	5	3	5	61.3%	16.1%	dissent	30	96.8%
16d	Percentage of Oncology centers participating in the care program	6.5%	12.9%	19.4%	19.4%	41.9%	3.77	±1.309	4	3	5	61.3%	19.4%	dissent	30	96.8%
17a	physicians staff turnover	0.0%	16.1%	22.6%	19.4%	41.9%	3.87	±1.147	4	3	5	61.3%	16.1%	dissent	31	100.0%
17b	Oncology centers staff turnover	3.2%	12.9%	19.4%	19.4%	45.2%	3.9	±1.221	4	3	5	64.6%	16.1%	dissent	30	96.8%
18a	Satisfaction of the service providers	0.0%	3.2%	3.2%	35.5%	58.1%	4.48	±0.724	5	4	5	93.6%	3.2%	strong (++)	30	96.8%
18b	Dissatisfaction of the service providers	3.2%	3.2%	3.2%	32.3%	58.1%	4.39	±0.955	5	4	5	90.4%	6.4%	strong (++)	30	96.8%
19	Participation in a service provider survey (response rate)	3.2%	3.2%	12.9%	41.9%	38.7%	4.1	±0.978	4	4	5	80.6%	6.4%	moderate (+)	30	96.8%
20	Communication in the nFC-isPO regarding service provider	0.0%	3.2%	9.7%	32.3%	54.8%	4.39	±0.803	5	4	5	87.1%	3.2%	moderate (+)	30	96.8%
21	Advanced training on patient-centered communication for service provider	3.2%	12.9%	22.6%	41.9%	19.4%	3.61	±1.054	4	3	4	61.3%	16.1%	dissent	30	96.8%
22	Trainings completed	0.0%	12.9%	29.0%	29.0%	29.0%	3.74	±1.032	4	3	5	58.0%	12.9%	dissent	29	93.5%
23	Participation in quality circles	0.0%	12.9%	19.4%	35.5%	32.3%	3.87	±1.024	4	3	5	67.8%	12.9%	dissent	29	93.5%
24	Organization of the quality circles	0.0%	12.9%	22.6%	38.7%	25.8%	3.77	±0.99	4	3	5	64.5%	12.9%	dissent	31	100.0%
25a	Percentage of total dropouts	0.0%	0.0%	13.8%	13.8%	72.4%	4.59	±0.733	5	4	5	86.2%	0.0%	moderate (+)	28	96.6%
25b	Percentage of dropouts - treatment contract revoked	0.0%	3.4%	27.6%	13.8%	55.2%	4.21	±0.978	5	3	5	69.0%	3.4%	dissent	28	96.6%
25c	Percentage of dropouts - change of health insurance	10.3%	10.3%	20.7%	24.1%	34.5%	3.62	±1.347	4	3	5	58.6%	20.6%	dissent	29	100.0%
25d	Percentage of dropouts - repeated non-appearance	0.0%	10.3%	37.9%	13.8%	37.9%	3.79	±1.082	4	3	5	51.7%	10.3%	dissent	29	100.0%
25e	Percentage of dropouts - deceased	3.4%	10.3%	24.1%	24.1%	37.9%	3.83	±1.167	4	3	5	62.0%	13.7%	dissent	29	100.0%

25f	Percentage of dropouts - complete takeover of another facility	6.9%	10.3%	17.2%	27.6%	37.9%	3.79	±1.264	4	3	5	65.5%	17.2%	dissent	29	100.0%
26a	Average time between enrolment and initial psychotherapeutic consultation	0.0%	0.0%	3.4%	27.6%	69.0%	4.66	±0.553	5	4	5	96.6%	0.0%	strong (++)	27	93.1%
26b	Average time between enrolment and initial psychosocial consultation	0.0%	3.4%	3.4%	27.6%	65.5%	4.55	±0.736	5	4	5	93.1%	3.4%	strong (++)	28	96.6%
26c	Average time between the appointment allocation and psychotherapeutic consultation	0.0%	10.3%	20.7%	10.3%	58.6%	4.17	±1.104	5	3	5	68.9%	10.3%	dissent	28	96.6%
26d	Average time between the appointment allocation and psychosocial consultation	0.0%	13.8%	20.7%	13.8%	51.7%	4.03	±1.149	5	3	5	65.5%	13.8%	dissent	28	96.6%
26e	Average time between psychotherapeutic consultation	0.0%	3.4%	24.1%	34.5%	37.9%	4.07	±0.884	4	3	5	72.4%	3.4%	dissent	28	96.6%
26f	Average time between psychosocial consultation	0.0%	10.3%	20.7%	37.9%	31.0%	3.9	±0.976	4	3	5	68.9%	10.3%	dissent	27	96.4%
27	Average time between initial diagnosis enrolment	0.0%	6.9%	13.8%	17.2%	62.1%	4.34	±0.974	5	4	5	79.3%	6.9%	moderate (+)	28	96.6%
28a	Number of initial consultations	0.0%	6.9%	24.1%	24.1%	44.8%	4.07	±0.998	4	3	5	68.9%	6.9%	dissent	25	86.2%
28b	Number of progress consultations	0.0%	3.4%	20.7%	27.6%	48.3%	4.21	±0.902	4	3.5	5	75.9%	3.4%	moderate (+)	25	86.2%
28c	Number of final consultations	0.0%	3.4%	34.5%	20.7%	41.4%	4	±0.964	4	3	5	62.1%	3.4%	dissent	25	86.2%
28d	Number of psychotherapeutic consultations	0.0%	6.9%	17.2%	24.1%	51.7%	4.21	±0.978	5	3.5	5	75.8%	6.9%	moderate (+)	28	96.6%
28e	Number of psychosocial consultations	0.0%	6.9%	17.2%	27.6%	48.3%	4.17	±0.966	4	3.5	5	75.9%	6.9%	moderate (+)	28	96.6%
28f	Number of relatives' consultations	17.2%	10.3%	17.2%	24.1%	31.0%	3.41	±1.476	4	2	5	55.1%	27.5%	dissent	26	89.7%
28g	Number of isPO-onco-guide consultations	0.0%	10.3%	24.1%	31.0%	34.5%	3.9	±1.012	4	3	5	65.5%	10.3%	dissent	26	89.7%
29a	Average number of psychotherapeutic consultations (step 3)	0.0%	10.7%	17.9%	10.7%	60.7%	4.21	±1.101	5	3	5	71.4%	10.7%	dissent	24	85.7%
29b	Percentage of patients counting 13 psychotherapeutic consultations (step 3)	3.6%	14.3%	28.6%	17.9%	35.7%	3.68	±1.219	4	3	5	53.6%	17.9%	dissent	26	92.9%

29c	Average number of psychosocial consultations (step 2)	0.0%	10.7%	17.9%	21.4%	50.0%	4.11	±1.066	4.5	3	5	71.4%	10.7%	dissent	25	89.3%
29d	Percentage of patients counting 6 psychosocial consultations (step 2)	3.6%	14.3%	32.1%	14.3%	35.7%	3.64	±1.224	3.5	3	5	50.0%	17.9%	dissent	26	92.9%
30a	Average duration of psychotherapeutic consultation	0.0%	7.4%	14.8%	25.9%	51.9%	4.22	±0.974	5	4	5	77.8%	7.4%	moderate (+)	27	100.0%
30b	Average duration of psychosocial consultation	0.0%	7.4%	18.5%	25.9%	48.1%	4.15	±0.989	4	3	5	74.0%	7.4%	dissent	27	100.0%
31	Percentage of complete documentation	0.0%	0.0%	0.0%	0.0%	0.0%	.	.	.	.	.	0.0%	0.0%	dissent	26	96.3%
32a	Average time between enrolment and documentation	0.0%	14.8%	18.5%	40.7%	25.9%	3.78	±1.013	4	3	5	66.6%	14.8%	dissent	26	96.3%
32b	Average time between data collection immediately after diagnosis (T1) and documentation	3.7%	14.8%	14.8%	40.7%	25.9%	3.7	±1.137	4	3	5	66.6%	18.5%	dissent	27	100.0%
32c	Average time between data collection 4 month after diagnosis (T2) and documentation	3.7%	18.5%	25.9%	40.7%	11.1%	3.37	±1.043	4	3	4	51.8%	22.2%	dissent	26	96.3%
32d	Average time between data collection 12 month after diagnosis (T3) and documentation	3.7%	18.5%	29.6%	37.0%	11.1%	3.33	±1.038	3	3	4	48.1%	22.2%	dissent	26	96.3%
32e	Average time between all data collections (T1, T2, T3) and documentation	3.7%	22.2%	22.2%	44.4%	7.4%	3.3	±1.031	4	2	4	51.8%	25.9%	dissent	25	92.6%
33a	Level allocation in the nFC-isPO (step 1)	3.7%	7.4%	11.1%	3.7%	74.1%	4.37	±1.182	5	4	5	77.8%	11.1%	moderate (+)	25	92.6%
33b	Level allocation in the nFC-isPO (step 2)	0.0%	3.7%	14.8%	7.4%	74.1%	4.52	±0.893	5	4	5	81.5%	3.7%	moderate (+)	26	96.3%
33c	Level allocation in the nFC-isPO (step 3a)	0.0%	3.7%	11.1%	11.1%	74.1%	4.56	±0.847	5	4	5	85.2%	3.7%	moderate (+)	26	96.3%
33d	Level allocation in the nFC-isPO (step 3b)	0.0%	3.7%	11.1%	7.4%	77.8%	4.59	±0.844	5	5	5	85.2%	3.7%	moderate (+)	26	96.3%
34a	Type of treatment (step 3) - individual psychotherapy	3.7%	14.8%	29.6%	14.8%	37.0%	3.67	±1.24	4	3	5	51.8%	18.5%	dissent	25	92.6%
34b	Type of treatment (step 3) - psychotherapeutic group therapy	11.1%	14.8%	37.0%	11.1%	25.9%	3.26	±1.318	3	2	5	37.0%	25.9%	dissent	25	92.6%

34c	Type of treatment (step 3) - pharmacotherapy for anxiety and depression	3.7%	3.7%	33.3%	29.6%	29.6%	3.78	±1.05	4	3	5	59.2%	7.4%	dissent	24	88.9%
34d	Type of treatment (step 3) - complex therapy consisting of psychotherapy and psychosocial counseling	3.7%	7.4%	18.5%	14.8%	55.6%	4.11	±1.188	5	3	5	70.4%	11.1%	dissent	26	96.3%
35	Percentage of self-management and assistance planning based psychosocial care (step 2)	0.0%	7.4%	18.5%	29.6%	44.4%	4.11	±0.974	4	3	5	74.0%	7.4%	dissent	25	92.6%
36	Average time of pharmacotherapy for anxiety and depression	7.4%	7.4%	22.2%	25.9%	37.0%	3.78	±1.251	4	3	5	62.9%	14.8%	dissent	23	85.2%
37a	Severity of depression - confirmed diagnosis of mild depression	3.7%	7.4%	14.8%	25.9%	48.1%	4.07	±1.141	4	3	5	74.0%	11.1%	dissent	25	92.6%
37b	Severity of depression - confirmed diagnosis of moderate depression	3.7%	3.7%	22.2%	25.9%	44.4%	4.04	±1.091	4	3	5	70.3%	7.4%	dissent	25	92.6%
37c	Severity of depression - confirmed diagnosis of major depression	3.7%	7.4%	14.8%	29.6%	44.4%	4.04	±1.126	4	3	5	74.0%	11.1%	dissent	25	92.6%
38a	Improvement rates - improvement in anxiety and depression at the end of the treatment period compared to the start of treatment	0.0%	0.0%	3.7%	14.8%	81.5%	4.78	±0.506	5	5	5	96.3%	0.0%	strong (++)	25	92.6%
38b	Improvement rates – response to treatment 4 months (T2) after treatment initiation	0.0%	0.0%	11.1%	11.1%	77.8%	4.67	±0.679	5	5	5	88.9%	0.0%	moderate (+)	23	85.2%
38c	Improvement rates – response to treatment 12 months (T3) after treatment initiation	0.0%	0.0%	7.4%	7.4%	85.2%	4.78	±0.577	5	5	5	92.6%	0.0%	strong (++)	24	88.9%
38d	Improvement rates – remission of anxiety and depression 4 months (T2) after treatment initiation	0.0%	3.7%	14.8%	11.1%	70.4%	4.48	±0.893	5	4	5	81.5%	3.7%	moderate (+)	24	88.9%
38e	Improvement rates – remission of anxiety and depression 12 months (T3) after treatment initiation	0.0%	0.0%	7.4%	14.8%	77.8%	4.7	±0.609	5	5	5	92.6%	0.0%	strong (++)	25	92.6%

39	Inpatient admission	18.5%	0.0%	14.8%	25.9%	40.7%	3.7	±1.489	4	3	5	66.6%	18.5%	dissent	24	88.9%
40	Suicidal assessment	7.4%	7.4%	11.1%	18.5%	55.6%	4.07	±1.299	5	3	5	74.1%	14.8%	dissent	27	100.0%
41a	completed suicides	3.7%	7.4%	22.2%	22.2%	44.4%	3.96	±1.16	4	3	5	66.6%	11.1%	dissent	25	92.6%
41b	Suicide attempts	3.7%	7.4%	18.5%	22.2%	48.1%	4.04	±1.16	4	3	5	70.3%	11.1%	dissent	26	96.3%
42	Treatment continuity for suicidality	11.1%	3.7%	18.5%	18.5%	48.1%	3.89	±1.368	4	3	5	66.6%	14.8%	dissent	25	92.6%

Threshold for consensus: "strong consensus (++)" ≥ 90% in category 5 and 4; "moderate consensus (+)" ≥ 75% in category 5 and 4; "strong rejection (--)" ≥ 90% in category 2 and 1; "moderate rejection (-)" ≥ 75% in category 2 and 1; dissent: no unanimous group response.
